# lncRNA PVT1: a novel oncogene in multiple cancers

**DOI:** 10.1186/s11658-022-00385-x

**Published:** 2022-10-04

**Authors:** Ruiming Li, Xia Wang, Chunming Zhu, Kefeng Wang

**Affiliations:** 1 Department of Urology, Shengjing Hospital of China Medical University, #36 Sanhao Street, Heping District, Shenyang, 110004 Liaoning China; 2 Department of Family Medicine, Shengjing Hospital of China Medical University, #36 Sanhao Street, Heping District, Shenyang, 110004 Liaoning China

**Keywords:** Long  noncoding RNA, PVT1, Oncogene, Cancer

## Abstract

Long noncoding RNAs are involved in epigenetic gene modification, including binding to the chromatin rearrangement complex in pre-transcriptional regulation and to gene promoters in gene expression regulation, as well as acting as microRNA sponges to control messenger RNA levels in post-transcriptional regulation. An increasing number of studies have found that long noncoding RNA plasmacytoma variant translocation 1 (PVT1) plays an important role in cancer development. In this review of a large number of studies on PVT1, we found that PVT1 is closely related to tumor onset, proliferation, invasion, epithelial–mesenchymal transformation, and apoptosis, as well as poor prognosis and radiotherapy and chemotherapy resistance in some cancers. This review comprehensively describes PVT1 expression in various cancers and presents novel approaches to the diagnosis and treatment of cancer.

## Introduction

Cancer death rate has continuously declined in the past 2 years, although it remains the second leading cause of death worldwide according to the latest cancer statistics from the USA [1]. Changes at the gene level include gene mutations, amplification, deletion, DNA methylation, and insertion mutations. Abnormal oncogene activation and tumor suppressor gene inactivation are important factors that lead to the occurrence of cancer. Advances in genetic research have revealed the pathogenesis of many cancers, which has led to the development of effective treatments. For example, leukemia caused by breakpoint cluster region–Abelson mutations can be effectively cured using imatinib [2]. Protein-coding genes account for < 2% of the total genome. The remaining genome consists of noncoding genes that are responsible for the vast majority of tumorigenesis.

Long noncoding RNAs (lncRNAs) are RNAs that are longer than 200 nucleotides. lncRNAs are located in the nucleus or cytoplasm of eukaryotic cells and have a variety of functions, including regulating gene expression in both a *cis* and a *trans* manner, acting as a sponge for microRNA (miRNA), and directly affecting proteins and other RNAs [3]. In the early twentieth century, the lncRNA X-inactive specific transcript was first discovered, wrapping the X chromosome in a *ci*s-like manner, resulting in silencing of the entire chromosome. lncRNAs have been discovered and have been proven to play important roles in many diseases and cell development and as biomarkers. For example, abnormal MALAT1 expression promotes the development of bladder cancer [4]. lncRNAs in kinase activation are associated with poor prognosis in patients with breast or lung cancer [5]. However, the mechanism by which lncRNAs regulate tumor development remains unclear, and further studies are needed to explore and summarize the potential mechanisms of action of lncRNAs.

The lncRNA plasmacytoma variant translocation 1 (PVT1), which is located on the telomeres of chromosome 8 in the c-Myc gene, was first reported by Guan et al. [5]. Recently, abnormal PVT1 expression was found in a variety of human malignancies, including non-small cell lung cancer (NSCLC) [6], nasopharyngeal carcinoma (NPC) [7], gastric cancer (GC) [8], oral squamous cell carcinoma (OSCC) [9], esophageal cancer (EC) [10], colorectal cancer (CRC) [11], hepatocellular carcinoma (HCC) [12], gallbladder cancer (GBC) [13], cholangiocarcinoma (CCA) [14], pancreatic cancer (PC) [15], breast cancer (BC) [16], cervical cancer (CC) [17], endometrial cancer [18], ovarian cancer (OC) [19], renal cell carcinoma (RCC) [20], bladder cancer [21], prostate cancer (PCa) [22], glioma [23], osteosarcoma (OS) [24], multiple myeloma (MM) [25], thyroid cancer (TC) [26], lymphoma [27], and leukemia [28]. Identifying the mechanisms of action of various cancers has helped researchers discover new biomarkers and therapeutic targets. Here, we aimed to identify new biomarkers and therapeutic targets by summarizing the mechanisms of PVT1 in various cancers.

## Functions of lncRNA

Many studies have shown that lncRNAs play vital roles as biomarkers in cancer diagnosis, therapeutic targets, prognosis, drug sensitizers, stratification, and other aspects [6–11].

lncRNAs can regulate chromatin topology by binding to H3K4me3 and H3K36me3 and through histone acetylation and other activated histone modifications to promote gene transcription [29]. Conversely, lncRNAs induce chromatin closure by binding to H3K9me3, H3K27me3, and H4K20me3 and through DNA methylation and other suppressive histone modification factors [30]. lncRNAs can serve as a central platform for assembling relevant molecular components, binding to nucleic acids through sequence complementation, and binding to proteins through RNA structural elements [31–33]. A variety of signaling pathways rely on scaffolds to deliver precise signals. lncRNAs can bind to multiple effector elements in the same space at the same time and integrate the information of the depression effector elements or activity effector elements. lncRNAs act as decoys and can directly bind to proteins, messenger RNAs (mRNAs), and miRNAs [34–36]. Accumulating evidence suggests that the decoy function of lncRNAs plays an irreplaceable role in tumor initiation, progression, and metastasis.

## Role of PVT1 in various human tumors

PVT1 is abnormally overexpressed in a variety of cancers and often promotes the occurrence, progression, invasion, metastasis, and chemoradiotherapy resistance in tumors by interacting with c-Myc [37–40]. The most common mechanism of PVT1 in various cancers is regulation of the relevant signaling pathways by competitive endogenous RNA (ceRNA), which promotes the occurrence and development of cancer. PVT1 can also directly regulate RNA, DNA, and protein expression. For example, in NPC, PVT1 stabilizes the structure of c-Myc by preventing its phosphorylation, promoting DNA repair in NPC cells, and increasing radiotherapy resistance [7]. In addition, PVT1 acts as a scaffold to regulate downstream genes and pathways. For example, in NPC, PVT1 promotes H3K9 acetylation by recruiting KAT2A [41]. PVT1 can recruit EZH2 to the promoter region of LAST2 and inhibit LAST2 expression in patients with NSCLC [42]. Similarly, PVT1 regulates P15/P16 and vascular endothelial growth factor (VEGF)A expression by recruiting EZH2 and signal transducer and activator of transcription (STAT)3 in GC cells [43]. In addition, PVT1 recruits EZH2 to downregulate miR-214 and P53 expression in HCC cells [44]. PVT1 regulates EZH2 binding to ANGPTL4 in CCA cells to inactivate its promoter region and reduce its expression [45]. In CC cells, PVT1 reduces the miR-200b expression by recruiting EZH2 to the promoter region of miR-200b [46]. In androgen-independent castration-resistant PCa, the promoter region of NOV is bound by EZH2 recruited by PVT1, which ultimately inhibits NOV expression [47]. In TC, PVT1 also promotes the proliferation of TC cells by recruiting EZH2, thereby affecting its expression of TSHR [26]. In general, PVT1 overexpression is associated with poor prognosis [48–51]. However, in the early stages of some cancers, such as OC, it predicts a good prognosis [52]. Recently, several studies have shown that PVT1 is involved in the clinical and pathological development of various cancers. The functions and mechanisms of action of PVT1 in human tumors are listed in Tables 1, 2, 3, 4, 5, 6, 7, 8, 9, 10, 11 and 12.

**Table 1:** Functional characterization of PVT1 in respiratory system tumors

Tumor type	Expression	Role	Function role	miRNAs	Related genes	References
Nasopharyngeal carcinoma	Upregulation	Oncogene	Proliferation	miR-1207	PI3K/AKT	[54]
Nasopharyngeal carcinoma	Upregulation	Oncogene	Proliferation and radioresistance	miR-515-5p	PIK3CA	[55]
Lung cancer	Upregulation	Oncogene	Proliferation	/	P15/P21	[6]
Lung cancer	Upregulation	Oncogene	Invasion and proliferation	miR-497	/	[56]
Lung cancer	Upregulation	Oncogene	Proliferation	/	LATS2	[42]
Lung cancer	Upregulation	Oncogene	Proliferation, migration, invasion, and EMT	miR-497	YAP1	[57]
Lung cancer	Upregulation	Oncogene	Proliferation and migration	miR-526b	EZH2	[58]
Lung cancer	Upregulation	Oncogene	Invasion	miR-200a/200b	MMP9	[59]
Lung cancer	Upregulation	Oncogene	Proliferation	miR-199a-5p	HIF-1α	[60]
Lung cancer	Upregulation	Oncogene	Angiogenesis	miR-29c	VEGF	[61]
Lung cancer	Upregulation	Oncogene	Proliferation and metastasis	miR-128	VEGFC	[62]
Lung cancer	Upregulation	Oncogene	Proliferation, migration, and invasion	miR-361-3p	SOX9	[63]
Lung cancer	Upregulation	Oncogene	Migration and invasion	miR-760	IL-6	[64]
Lung cancer	Upregulation	Oncogene	Proliferation	miR-17-5p	BAMBI	[65]
Lung cancer	Upregulation	Oncogene	Proliferation and migration	miR-148	RAB34	[66]
Lung cancer	Upregulation	Oncogene	Proliferation, migration, and invasion	miR-551b	FGFR1	[67]
Lung cancer	Upregulation	Oncogene	Invasion and growth	miR-378c	SLC2A1	[68]
Lung cancer	Upregulation	Oncogene	Radioresistance	miR-195	/	[69]
Lung cancer	Upregulation	Oncogene	Radioresistance	miR-424-5p	CARM1	[70]
Lung cancer	Upregulation	Oncogene	Cisplatin-resistance	miR-181a-5p	SP1	[71]
Lung cancer	Upregulation	Oncogene	Cisplatin-resistance	miR-216b	Beclin-1	[72]

**Table 2:** Main characteristics of the studies included in the review of respiratory system tumors

Study	Tumor type	Sample size (normal: tumor)	Detection method	*P* value	TNM (*P* value)	LNM (*P* value)	DM (*P* value)	OS (*P* value)	DFS (*P* value)	References
Cui	Nasopharyngeal carcinoma	15:15	qRT-PCR	< 0.001	/	/	/	0.0385	0.0261	[54]
Cui	Lung cancer	108:108	qRT-PCR	< 0.05	0.003	< 0.001	0.571	0.014	0.036	[6]
Wan	Lung cancer	105:105	qRT-PCR	< 0.05	0.001	0.011	/	/	/	[42]
Chen	Lung cancer	80:80	qRT-PCR	< 0.0001	/	/	/	/	/	[59]
Qi	Lung cancer	30:30	qRT-PCR	< 0.05	/	/	/	/	/	[63]
Wu	Lung cancer	31:31	qRT-PCR	< 0.05	0.017	0.018	/	/	/	[69]
Chen	Lung cancer	40:40	qRT-PCR	< 0.05	0.01	0.0146	/	/	/	[72]

**Table 3:** Functional characterization of PVT1 in digestive system tumors

Tumor type	Expression	Role	Function role	miRNAs	Related genes	References
Oral squamous cell carcinoma	Upregulation	Oncogene	Proliferation, invasion, and migration	miR-150-5p	GLUT-1	[9]
Oral squamous cell carcinoma	Upregulation	Oncogene	Proliferation and chemoresistance	miR-194-5p	HIF1a	[75]
Esophageal cancer	Upregulation	Oncogene	Proliferation, migration, and invasion	miR-186-5p	PTTG1	[10]
Esophageal cancer	Upregulation	Oncogene	Growth and invasion	miR-203	LASP1	[78]
Esophageal cancer	Upregulation	Oncogene	Migration and invasion	miR-145	FSCN1	[79]
Gastric cancer	Upregulation	Oncogene	Growth and invasion	/	FOXM1	[84]
Gastric cancer	Upregulation	Oncogene	Angiogenesis	/	Slug/VEGFA	[43]
Gastric cancer	Upregulation	Oncogene	Growth and invasion	miR-186	HIF-1α	[85]
Gastric cancer	Upregulation	Oncogene	Growth and invasion	miR-152	FGF2/CD151	[86]
Gastric cancer	Upregulation	Oncogene	EMT	miR-30a	Snail	[87]
Liver cancer	Upregulation	Oncogene	Proliferation	/	NOP2	[92]
Liver cancer	Upregulation	Oncogene	Growth and invasion	miR-214	/	[44]
Liver cancer	Upregulation	Oncogene	Proliferation	/	P53	[95]
Liver cancer	Upregulation	Oncogene	Migration and invasion	miR-3619-5p	MKL1	[97]
Liver cancer	Upregulation	Oncogene	Invasion and migration	miR-186-5p	YAP1	[98]
Liver cancer	Upregulation	Oncogene	Growth and invasion	miR-150	HIG2	[99]
Liver cancer	Upregulation	Oncogene	Proliferation	miR-424-5p	INCENP	[100]
Liver cancer	Upregulation	Oncogene	Autophagy	miR-365	ATG3	[101]
Cholangiocarcinoma	Upregulation	Oncogene	Proliferation, migration, and invasion	miR-186	SEMA4D	[14]
Cholangiocarcinoma	Upregulation	Oncogene	Migration and invasion	/	ANGPTL4	[45]
Gallbladder cancer	Upregulation	Oncogene	Proliferation	miR-18b-5p	HIF1A	[13]
Gallbladder cancer	Upregulation	Oncogene	Proliferation, migration, and invasion	miR-143	HK2	[105]
Gallbladder cancer	Upregulation	Oncogene	Growth and invasion	miR-30d-5p	/	[106]
Pancreatic cancer	Upregulation	Oncogene	EMT	/	TGF-β/Smad	[108]
Pancreatic cancer	Upregulation	Oncogene	EMT	/	P21	[109]
Pancreatic cancer	Upregulation	Oncogene	Proliferation and migration	miR-448	SERBP1	[110]
Pancreatic cancer	Upregulation	Oncogene	Progression and glycolysis	miR-519d-3p	HIF-1A	[111]
Pancreatic cancer	Upregulation	Oncogene	Autophagy	miR-20a-5p	ULK1	[113]
Pancreatic cancer	Upregulation	Oncogene	Chemoresistance	/	EZH2	[115]
Pancreatic cancer	Upregulation	Oncogene	Proliferation	miR-1207	/	[116]
Pancreatic cancer	Upregulation	Oncogene	Chemoresistance	miR-409	SHH/GLI/MGMT	[117]
Pancreatic cancer	Upregulation	Oncogene	Chemoresistance	miR-619-5p	Pygo2	[118]
Pancreatic cancer	Upregulation	Oncogene	Autophagy and chemoresistance	miR-143	HIF-1α/VMP1	[119]
Colorectal cancer	Upregulation	Oncogene	Proliferation and metastasis	miR-26b	/	[11]
Colorectal cancer	Upregulation	Oncogene	Metastasis	miR-152-3p	VEGFA, EGFR	[125]
Colorectal cancer	Upregulation	Oncogene	EMT	miR-216a-5p	YBX1	[126]
Colorectal cancer	Upregulation	Oncogene	EMT	miR-16-5p	VEGFA	[127]
Colorectal cancer	Upregulation	Oncogene	EMT	miR-186	Twist1	[128]
Colorectal cancer	Upregulation	Oncogene	Proliferation, invasion, and migration	miR-455	RAF-1	[129]
Colorectal cancer	Upregulation	Oncogene	Growth and invasion	miR-30d-5p	RUNX2	[130]
Colorectal cancer	Upregulation	Oncogene	Invasion and growth	miR-214-3p	IRS1	[131]
Colorectal cancer	Upregulation	Oncogene	Apoptosis	miR-146a	COX2	[132]
Colorectal cancer	Upregulation	Oncogene	Proliferation, migration, and invasion	miR-106b-5p	FJX1	[133]
Colorectal cancer	Upregulation	Oncogene	Proliferation	miR-761	MAPK1	[134]

**Table 4:** Main characteristics of the studies included in the review of digestive system tumors

Study	Tumor type	Sample size (normal: tumor)	Detection method	*P* value	TNM (*P* value)	LNM (*P* value)	DM (*P* value)	OS (*P* value)	DFS (*P* value)	References
Li	Oral squamous cell carcinoma	70:70	qRT-PCR	< 0.01	< 0.05	/	< 0.01	/	/	[9]
Zheng	Esophageal cancer	77:77	qRT-PCR	0.002	0.009	/	/	/	/	[77]
Li	Esophageal cancer	104:104	qRT-PCR	0.024	0.001	0.597	/	0.005	0.011	[78]
Xu	Gastric cancer	190:190	qRT-PCR	< 0.001	/	/	/	/	/	[84]
Huang	Gastric cancer	68:68	qRT-PCR	< 0.05	0.022	0.005	/	/	/	[85]
Li	Gastric cancer	20:20	qRT-PCR	0.0004	/	/	/	/	/	[86]
Wang	Gastric cancer	42:42	qRT-PCR	< 0.01	< 0.05	< 0.01	/	/	/	[87]
Ding	Liver cancer	58:58	qRT-PCR	0.042	< 0.05	/	/	/	0.021	[12]
Wang	Liver cancer	89:89	qRT-PCR	< 0.05	0.007	/	/	0.0104	0.0043	[92]
Gou	Liver cancer	92:92	qRT-PCR	< 0.05	< 0.05	/	/	/	/	[44]
Guo	Liver cancer	121:121	qRT-PCR	< 0.05	0.010	/	/	/	/	[95]
Lan	Liver cancer	48:48	qRT-PCR	< 0.001	< 0.05	/	/	0.035	/	[98]
Yang	Liver cancer	80:80	qRT-PCR	< 0.05	0.041	/	/	/	/	[101]
Jin	Gallbladder cancer	55:55	qRT-PCR	< 0.001	< 0.011	< 0.032	/	< 0.001	/	[13]
Chen	Gallbladder cancer	20:20	qRT-PCR	< 0.05	< 0.001	/	0.047	< 0.05	/	[105]
Wu	Pancreatic cancer	30:20	qRT-PCR	< 0.01	< 0.05	< 0.05	/	/	/	[109]
Zhao	Pancreatic cancer	34:34	qRT-PCR	< 0.05	0.038	/	0.000	/	/	[110]
Sun	Pancreatic cancer	30:30	qRT-PCR	< 0.001	0.05	0.004	/	/	/	[111]
Huang	Pancreatic cancer	68:68	qRT-PCR	< 0.001	< 0.05	/	/	< 0.05	/	[113]
Zeng	Colorectal cancer	70:70	qRT-PCR	< 0.001	0.029	0.014	/	/	/	[126]
Wu	Colorectal cancer	72:72	qRT-PCR	< 0.05	< 0.05	< 0.01	< 0.05	/	/	[127]
Chai	Colorectal cancer	20:20	qRT-PCR	< 0.001	/	/	/	/	/	[129]
Yu	Colorectal cancer	60:60	qRT-PCR	< 0.05	0.040	0.006	/	/	< 0.05	[130]
Liu	Colorectal cancer	62:62	qRT-PCR	< 0.05	0.0001	0.005	0.002	/	/	[133]
Ping	Colorectal cancer	112:112	qRT-PCR	< 0.05	0.001	0.015	0.007	/	/	[136]

**Table 5:** Functional characterization of PVT1 in endocrine reproductive system tumors

Tumor type	Expression	Role	Function role	miRNAs	Related genes	References
Breast cancer	Upregulation	Oncogene	Proliferation	/	P21	[140]
Breast cancer	Upregulation	Oncogene	EMT	/	SOX2	[142]
Breast cancer	Upregulation	Oncogene	Migration	/	Claudin 4	[145]
Breast cancer	Upregulation	Oncogene	Proliferation	miR-1207-5p	STAT6	[146]
Breast cancer	Upregulation	Oncogene	EMT and proliferation	miR-1204	VDR	[147]
Breast cancer	Upregulation	Oncogene	Proliferation	miR-543	TRPS1	[148]
Breast cancer	Upregulation	Oncogene	Chemoresistance	/	Nrf2	[149]
Ovarian cancer	Upregulation	Oncogene	Growth and invasion	miR-133a	/	[151]
Ovarian cancer	Upregulation	Oncogene	Proliferation	miR-140	/	[152]
Ovarian cancer	Upregulation	Oncogene	Proliferation, migration, and invasion	miR-214	/	[153]
Ovarian cancer	Upregulation	Oncogene	Growth	/	P57	[154]
Ovarian cancer	Upregulation	Oncogene	Proliferation, migration, and invasion	miR-543	SERPINI1	[155]
Ovarian cancer	Upregulation	Oncogene	Chemoresistance	/	TGF-β, p-Smad4, Caspase-3	[19]
Ovarian cancer	Upregulation	Oncogene	Proliferation, migration, invasion, viability, and EMT	miR-148a-3p	AGO1	[156]
Ovarian cancer	Upregulation	Oncogene	Proliferation, migration, and invasion	miR-370	FOXM1	[158]
Ovarian cancer	Upregulation	Oncogene	Proliferation and invasion	/	JAK2/STAT3/PD-L1	[159]
Cervical cancer	Upregulation	Oncogene	Proliferation and migration	miR-200b	/	[46]
Cervical cancer	Upregulation	Oncogene	EMT and chemoresistance	miR-195	/	[163]
Cervical cancer	Upregulation	Oncogene	Proliferation, migration, and invasion	miR-424	/	[164]
Cervical cancer	Upregulation	Oncogene	Growth	/	TGF-β1	[165]
Cervical cancer	Upregulation	Oncogene	Growth and apoptosis	miR-16	NF-κB	[166]
Cervical cancer	Upregulation	Oncogene	Growth and invasion	miR-486-3p	ECM1	[167]
Cervical cancer	Upregulation	Oncogene	Proliferation, migration, and invasion	miR-503	ARL2	[168]
Cervical cancer	Upregulation	Oncogene	Growth and invasion	miR-140-5p	Smad3	[169]
Endometrial carcinoma	Upregulation	Oncogene	Proliferation, migration, and invasion	miR-195-5p	FGFR1/FGF2	[18]

**Table 6:** Main characteristics of the studies included in the review of endocrine reproductive system tumors

Study	Tumor type	Sample size (normal: tumor)	Detection method	*P* value	TNM (*P* value)	LNM (*P* value)	DM (*P* value)	OS (*P* value)	DFS (*P* value)	References
Li	Breast cancer	84:84	qRT-PCR	< 0.01	0.061	0.117	/	0.034	0.045	[140]
Wang	Breast cancer	38:38	qRT-PCR	< 0.0001	0.002	0.023	0.009	0.003	/	[142]
Wang	Breast cancer	30:30	qRT-PCR	< 0.05	/	/	/	/	/	[148]
Yang	Ovarian cancer	42:42	qRT-PCR	< 0.01	/	/	/	< 0.05	/	[151]
Ding	Ovarian cancer	/	qRT-PCR	< 0.05	< 0.05	/	/	0.0230	/	[152]
Chen	Ovarian cancer	58:231	qRT-PCR	< 0.05	< 0.05	0.436	/	0.020	0.016	[153]
Qu	Ovarian cancer	42:42	qRT-PCR	< 0.01	/	/	/	0.012	/	[155]
El-Khazragy	Ovarian cancer	30:100	qRT-PCR	< 0.001	0.001	/	0.001	0.001	0.01	[157]
Chen	Ovarian cancer	38:91	qRT-PCR	< 0.001	< 0.05	< 0.05	/	< 0.001	0.002	[159]
Iden	Cervical cancer	30:127	qRT-PCR	< 0.001	/	/	/	0.03	/	[17]
Zhang	Cervical cancer	90:90	qRT-PCR	< 0.001	/	/	/	0.015	/	[46]
Wang	Cervical cancer	39:156	qRT-PCR	< 0.05	/	/	/	< 0.05	/	[165]

**Table 7:** Functional characterization of PVT1 in urinary system tumors

Tumor type	Expression	Role	Function role	miRNAs	Related genes	References
Renal cell carcinoma	Upregulation	Oncogene	Apoptosis	/	EGFR/AKT/MYC	[173]
Renal cell carcinoma	Upregulation	Oncogene	Apoptosis	/	Mcl-1	[174]
Renal cell carcinoma	Upregulation	Oncogene	Proliferation, migration, invasion, and angiogenesis	/	HIF2α	[175]
Renal cell carcinoma	Upregulation	Oncogene	Proliferation and invasion	miR-200s	BMI1, ZEB1 and ZEB2	[179]
Renal cell carcinoma	Upregulation	Oncogene	Proliferation, invasion, and EMT	miR-16-5p	/	[180]
Bladder cancer	Upregulation	Oncogene	Proliferation, migration, and invasion	miR-31	CDK1	[184]
Bladder cancer	Upregulation	Oncogene	Proliferation and migration	miR-128	VEGFC	[185]
Bladder cancer	Upregulation	Oncogene	Proliferation,migration, and apoptosis	miR-194-5p	BCLAF1	[186]
Bladder cancer	Upregulation	Oncogene	Proliferation,invasion, apoptosis, and chemoresistance	/	Wnt/β-catenin	[187]
Prostate cancer	Upregulation	Oncogene	Growth	/	c-Myc	[189]
Prostate cancer	Upregulation	Oncogene	EMT	miR-186	Twist1	[190]
Prostate cancer	Upregulation	Oncogene	Proliferation and migration	/	P38	[191]
Prostate cancer	Upregulation	Oncogene	Metastasis	/	NOP2	[192]
Prostate cancer	Upregulation	Oncogene	Migration and invasion	miR-15a-5p	KIF23	[193]
Prostate cancer	Upregulation	Oncogene	Growth	miR-146a	/	[194]

**Table 8:** Main characteristics of the studies included in the review of urinary system tumors

Study	Tumor type	Sample size (normal: tumor)	Detection method	*P* value	TNM (*P* value)	LNM (*P* value)	DM (*P* value)	OS (*P* value)	DFS (*P* value)	References
Li	Renal cancer	40:40	qRT-PCR	< 0.001	< 0.05	/	/	< 0.01	/	[173]
Zhuang	Bladder cancer	32:32	qRT-PCR	< 0.01	0.002	/	/	/	/	[21]
Li	Bladder cancer	98:98	qRT-PCR	< 0.001	0.02	< 0.001	/	0.004	/	[183]
Tian	Bladder cancer	35:35	qRT-PCR	< 0.01	/	/	/	/	/	[184]
Chen	Bladder cancer	27:43	qRT-PCR	< 0.01	< 0.001	0.394	/	/	/	[186]
Yang	Prostate cancer	30:152	qRT-PCR	< 0.0001	< 0.01	> 0.05	/	0.0127	0.0097	[189]
Chang	Prostate cancer	52:499	qRT-PCR	< 0.05	/	/	/	0.046	/	[190]
Wu	Prostate cancer	25:25	qRT-PCR	< 0.01	/	/	/	/	/	[193]

**Table 9:** Functional characterization of PVT1 in immune system

Disease type	Expression	Role	Function role	miRNAs	Related genes	References
Sjogren’s syndrome	Upregulation	Pathogenic factor	Activate CD4^+^ T cells	/	/	[196]
Heart transplant rejection	Downregulation	Protection factor	Regulation of autophagy in regulatory T cells	miR-146a	/	[197]
Infectious myocardial injury	Upregulation	Pathogenic factor	Promote macrophage polarization	miR-29a	HMGB1	[198]
Immune thrombocytopenia	Downregulation	Protection factor	Downregulation of Th17 cell differentiation	/	NOTCH1	[199]
Tumor immune suppression	Upregulation	Pathogenic factor	Enhancing the function of MDSCs	/	/	[200]

**Table 10:** Main characteristics of the studies included on the immune system

Study	Disease type	Sample size (normal: tumor)	Detection method	*P* value	TNM (*P* value)	LNM (*P* value)	DM (*P* value)	OS (*P* value)	DFS (*P* value)	References
Fu	Sjogren’s syndrome	4:4	qRT-PCR	< 0.05	/	/	/	/	/	[196]
Lu	Transplant rejection	6:6	qRT-PCR	< 0.01	/	/	/	/	/	[197]
Yu	Immune thrombocytopenia	12:12	qRT-PCR	< 0.01	/	/	/	/	/	[199]

**Table 11:** Functional characterization of PVT1 in tumors of other systems

Tumor type	Expression	Role	Function role	miRNAs	Related genes	References
Glioma	Upregulation	Oncogene	Growth	miR-190a-5p/miR-488-3p	MEF2C	[202]
Glioma	Upregulation	Oncogene	Cell activity, migration, and invasion	miR-424	/	[203]
Glioma	Upregulation	Oncogene	Growth and invasion	miR-200a	/	[204]
Glioma	Upregulation	Oncogene	Proliferation and migration	/	UPF1	[205]
Glioma	Upregulation	Oncogene	Cell activity, growth, and invasion	miR-128-3p	GREM1	[23]
Glioma	Upregulation	Oncogene	Proliferation	miR-128-1-5p	PTBP1	[206]
Glioma	Upregulation	Oncogene	Proliferation, invasion, and apoptosis	miR-1301-3p	TMBIM6	[207]
Glioma	Upregulation	Oncogene	EMT	miR-1207-3p	HNF1B	[208]
Glioma	Upregulation	Oncogene	Proliferation and migration	miR-186	Atg7/Beclin1	[209]
Glioma	Upregulation	Oncogene	Proliferation, migration, invasion, stemness, and chemoresistance	miR-365	ELF4/SOX2	[210]
Osteosarcoma	Upregulation	Oncogene	Proliferation, migration, and invasion	miR-195	BCL2	[24]
Thyroid cancer	Upregulation	Oncogene	Proliferation	/	TSHR	[26]
Thyroid cancer	Upregulation	Oncogene	Viability and invasion	miR-30a	IGF1R	[212]
Thyroid cancer	Upregulation	Oncogene	Proliferating, invasion, and apoptosis	miR-423-5p	PAK3	[213]
Osteosarcoma	Upregulation	Oncogene	Growth and invasion	miR-497	HK2	[217]
Osteosarcoma	Upregulation	Oncogene	Growth and metastasis	miR-183-5p	ERG	[218]
Osteosarcoma	Upregulation	Oncogene	Metastasis	miR-484	/	[219]
Osteosarcoma	Upregulation	Oncogene	Chemoresistance	miR-152	c-MET/PI3K/AKT	[222]
Multiple myeloma	Upregulation	oncogene	Proliferation	miR-203a	/	[25]
Leukemia	Upregulation	Oncogene	Growth, migration, invasion, and apoptosis	miR-29 family	WAVE1	[231]

**Table 12:** Main characteristics of the studies included in the review of other system tumors

Study	Tumor type	Sample size (normal: tumor)	Detection method	*P* value	TNM (*P* value)	LNM (*P* value)	DM (*P* value)	OS (*P* value)	DFS (*P* value)	References
Gong	Glioma	28:30	qRT-PCR	< 0.01	< 0.01	/	/	0.0240	/	[210]
Zhou	Osteosarcoma	26:26	qRT-PCR	< 0.05	/	/	/	< 0.05	/	[24]
Song	Osteosarcoma	46:46	qRT-PCR	0.0009	< 0.001	/	/	0.011	/	[217]
Yan	Osteosarcoma	48:48	qRT-PCR	< 0.0001	0.008	/	0.001	0.0001	/	[219]
Xun	Osteosarcoma	78:78	qRT-PCR	< 0.001	0.000	0.017	0.017	/	/	[220]
Zhou	Thyroid cancer	84:84	qRT-PCR	< 0.0001	/	/	/	/	/	[26]
Feng	Thyroid cancer	72:72	qRT-PCR	< 0.05	< 0.01	< 0.01	/	/	/	[212]
Lin	Thyroid cancer	47:47	qRT-PCR	< 0.001	/	/	/	/	/	[213]
Yang	Multiple myeloma	33:14	qRT-PCR	< 0.05	/	/	/	/	/	[25]
Yang	Leukemia	62:286	qRT-PCR	< 0.001	/	/	/	< 0.001	/	[27]
Izadifard	AML	40:22	qRT-PCR	0.017	/	/	/	/	/	[227]
El-Khazragy	AML	70:20	qRT-PCR	< 0.01	/	/	/	/	/	[228]
Cheng	AML	30:30	qRT-PCR	< 0.01	/	/	/	/	/	[231]

### Role of PVT1 in the respiratory system

#### PVT1 in nasopharyngeal carcinoma

NPC originates from nasopharyngeal epithelial cells and is particularly common in Southeast Asia, North Africa, and southern China. New cases accounted for only 0.7% of the total cancer incidence, and the mortality rate has decreased significantly with advancements in medical treatments. However, the treatment of NPC is difficult owing to radiotherapy and chemotherapy resistance, postoperative recurrence, diffusion, and metastasis, which require further exploration to better diagnose and treat patients with NPC [53].

**Fig. 1:**
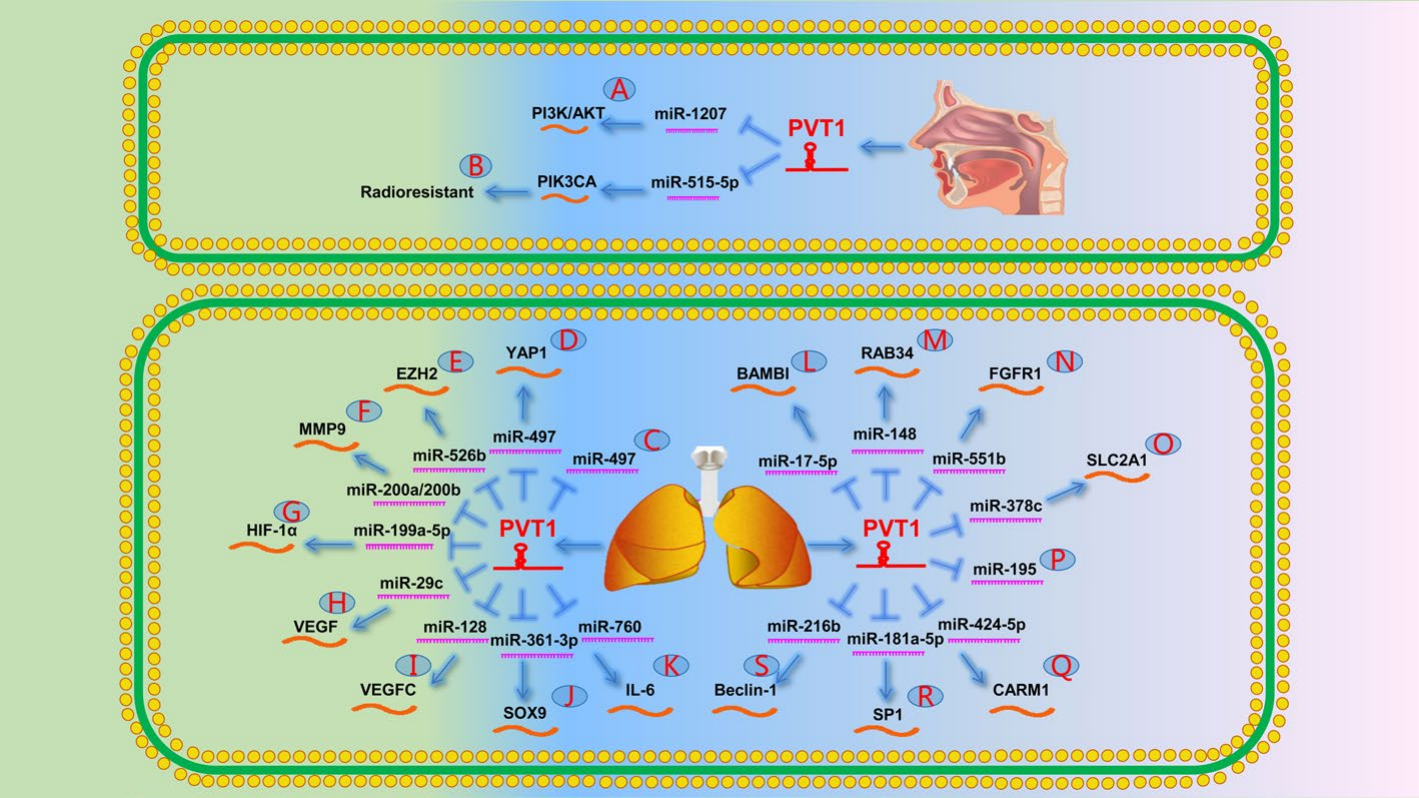
The role of PVT1 in the respiratory system. **A** PVT1 promotes the expression of PI3K/AKT by targeting miR-1207. **B** PVT1 promotes the expression of PIK3CA by targeting miR-515-5p, which in turn causes radioresistance. **C** PVT1 directly targets miR-497. **D** PVT1 promotes the expression of YAP1 by targeting miR-497. **E** PVT1 promotes the expression of EZH2 by targeting miR-526b. **F** PVT1 promotes the expression of MMP9 by targeting miR-200a/200b. **G** PVT1 promotes the expression of HIF-1α by targeting miR-199a-5p. **H** PVT1 promotes the expression of VEGF by targeting miR-29c. **I** PVT1 promotes the expression of VEGFC by targeting miR-128. **J** PVT1 promotes the expression of SOX9 by targeting miR-361-3p. **K** PVT1 promotes the expression of IL-6 by targeting miR-760. **L** PVT1 promotes the expression of BAMBI by targeting miR-17-5p. **M** PVT1 promotes the expression of RAB34 by targeting miR-148. **N** PVT1 promotes the expression of FGFR1 by targeting miR-551b. **O** PVT1 promotes the expression of SLC2A1 by targeting miR-378c. **P** PVT1 directly targets miR-195. **Q** PVT1 promotes the expression of CARM1 by targeting miR-424-5p. **R** PVT1 promotes the expression of SP1 by targeting miR-181a-5p. **S** PVT1 promotes the expression of Beclin-1 by targeting miR-216b

In a study by Cui et al. [54] on the formation, metastasis, and recurrence of NPC, the authors reported that PVT1 promoted tumor stem cell growth by inhibiting miR-1207 and activating the phosphatidylinositol 3-kinase/protein kinase B (PI3K/AKT) signaling pathway (Fig. 1A). In addition, He et al. [7] demonstrated that PVT1 acted as a radioresistant factor, thereby promoting DNA repair and inhibiting apoptosis in NPC cells by activating ATM/Chk2/p53 phosphorylation. Han et al. [55] revealed that PVT1 controls NPC cell radioresistance and proliferation through the miR-515-5p/PIK3CA axis (Fig. 1B). Wang et al. [41] discovered another mechanism by which PVT1 reduces radiotherapy sensitivity in NPC cells. They validated that PVT1 acts as a scaffold to promote H3K9 acetylation by the chromatin modifier KAT2A, thereby activating the downstream expression of nuclear factor 90 through H3K9ac binding to TIF1β. Furthermore, the stability of hypoxia-inducible factor (HIF)-1α mRNA was further strengthened to achieve tumor resistance to radiotherapy.

In conclusion, PVT1 plays an important role in the tumorigenesis and radioresistance of NPC.

#### PVT1 in non-small cell lung cancer

As of 2022, lung cancer accounted for 12% and 13% of all cancers in men and women, respectively [1]. Advanced NSCLC remains the leading cause of cancer-related deaths. However, little progress has been made in understanding its molecular mechanisms. Therefore, a study elucidating the underlying mechanisms of NSCLC is urgently needed.

A previous study showed that PVT1 promotes NSCLC cell proliferation by inhibiting P15 and P21 expression [6]. Another study reported that PVT1 regulates miR-497 to promote tumor formation in patients with NPC (Fig. 1C) [56]. Wan et al. [42] demonstrated that PVT1 repressed the expression of LAST2 by recruiting EZH2 to the promoter region of LAST2. LAST2 is an upstream phosphokinase of the Hippo signaling pathway that can phosphorylate YAP1 to reduce its expression and activate the Hippo signaling pathway. Subsequently, Zeng et al. [57] further confirmed that PVT1 promoted YAP1 expression by downregulating miR-497 by mediating EZH2, leading to the inhibition of the Hippo signaling pathway. Ultimately, the neurogenic locus notch homolog protein 1 (NOTCH1) signaling pathway was activated to promote NSCLC cell proliferation, migration, and epithelial–mesenchymal transition (EMT) (Fig. 1D). Another study emphasized that PVT1 upregulated EZH2 by acting as a sponge for miR-526b (Fig. 1E) [58].

PVT1 can also promote NSCLC progression as a ceRNA. Chen et al. [59] showed that PVT1 overexpression promoted NSCLC invasion by competitively binding miR-200a/200b to upregulate MMP9 expression (Fig. 1F). Additionally, Wang et al. [60] indicated that PVT1 was overexpressed in patients with hypoxic NSCLC and upregulated HIF-1α expression by acting as a sponge for miR-199a-5p. These findings suggest that PVT1 may serve as a potential therapeutic target for the treatment of hypoxic NSCLC (Fig. 1G). Several studies have indicated an important role of the lncRNA–miRNA–mRNA axis in patients with NSCLC. Specifically, PVT1 reportedly contributed to the proliferation, migration, invasion, and apoptosis of NSCLC cells by upregulating VEGF, VEGFC, SOX9, interleukin-6, BAMBI, RAB34, fibroblast growth factor receptor 1 (FGFR1), and SLC2A1 via miR-29c, miR-128, miR-361-3p, miR-760, miR-17-5p, miR-148, miR-551b, and miR-378c regulation (Fig. 1H−O) [61–68], respectively.

PVT1 also plays an important role in radiotherapy and chemotherapy in patients with advanced NSCLC. Wu et al. [69] found that miR-195 release after PVT1 knockdown increased NSCLC cell apoptosis during radiotherapy (Fig. 1P). Subsequently, Wang et al. [70] revealed that PVT1 and CARM1 downregulation led to miR-424-5p overexpression, which may improve radiosensitivity in patients with NSCLC (Fig. 1Q). Interestingly, the Chinese herbal prescription Xiaoji decoction enhanced the efficacy of cisplatin in patients with NSCLC by decreasing the expression of the PVT1/miR-181a-5p/SP1 axis (Fig. 1R) [71]. Similarly, Chen et al. [72] reported that PVT1 enhances cisplatin resistance in patients with NSCLC by regulating the miR-216b/Beclin-1 axis (Fig. 1S).

These results suggest that lncRNA PVT1 plays a vital role in the invasion, proliferation, migration, drug resistance, and radiosensitivity of NSCLC cells.

### Role of PVT1 in the digestive system

#### PVT1 in oral squamous cell carcinoma

OSCC is a malignant tumor derived from oral epithelial cells with a high incidence in Southeast Asia and southern China that may be related to dietary habits, such as betel nut consumption. In 2021, there were 377,713 new cases, accounting for 2% of all cancers, and 177,757 new deaths accounting for 1.8% of all cancers [73]. The number of patients with OSCC is expected to increase by 30% within the next 10 years, with a 5-year survival rate of only 50% [53]. Therefore, further studies on the pathogenesis of OSCC are required to improve its diagnosis, treatment, and prognosis.

**Fig. 2:**
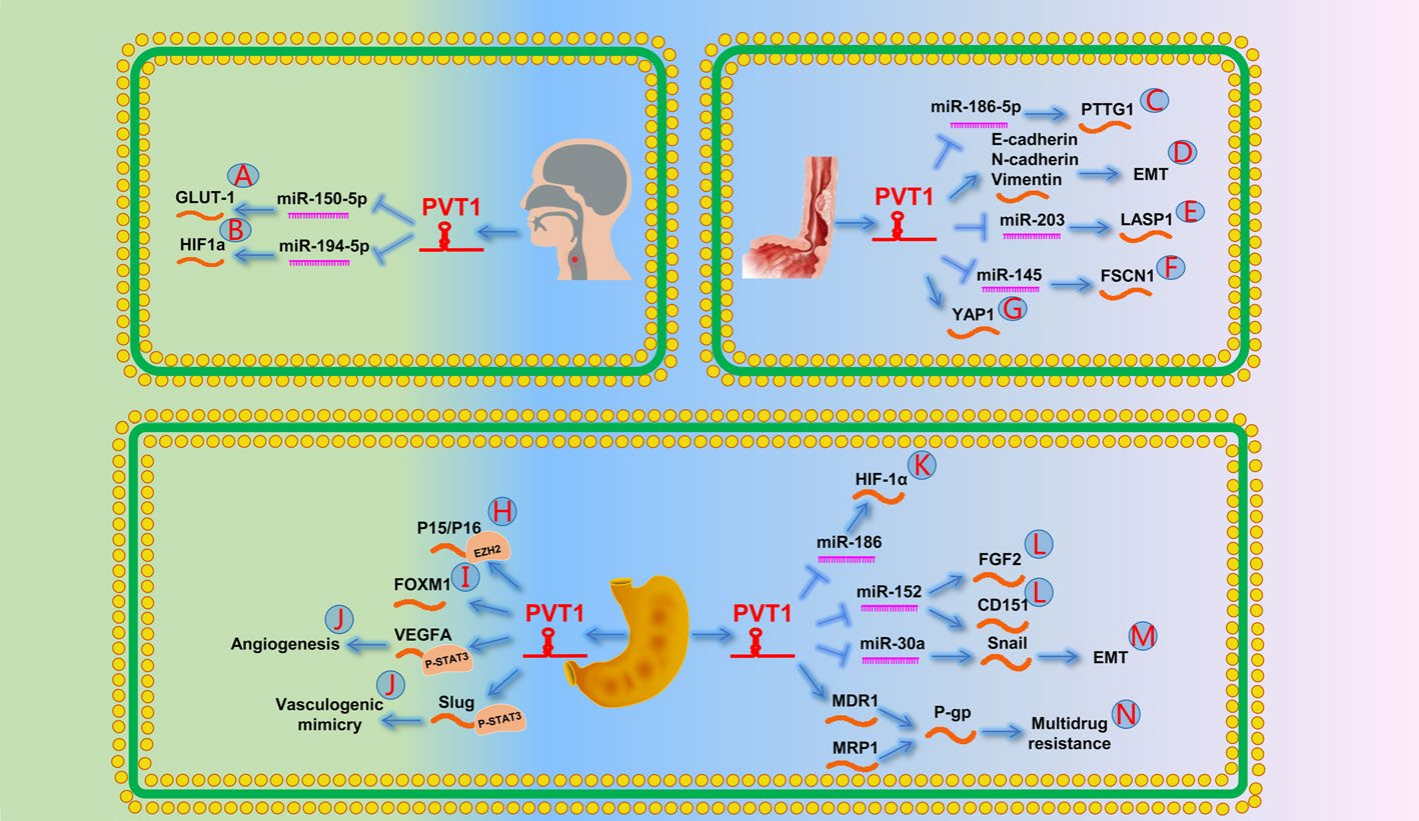
The role of PVT1 in the digestive system [1]. **A** PVT1 promotes the expression of GLUT-1 by targeting miR-150-5p. **B** PVT1 promotes HIF1a by targeting miR-194-5p and leads to cisplatin resistance in OSCC. **C** PVT1 promotes the expression of PTTG1 by targeting miR-186-5p. **D** PVT1 can promote the expression of EMT markers (E-cadherin, N-cadherin, and vimentin) in EC cells, indicating that it can promote the occurrence of EMT. **E** PVT1 promotes the expression of LASP1 by targeting miR-203. **F** PVT1 promotes the expression of FSCN1 by targeting miR-145. **G** PVT1 prevents the inactivation of Hippo’s downstream effector YAP1 by reducing its phosphorylation. **H** PVT1 downregulates P15/P16 expression by recruiting EZH2. **I** A positive feedback loop between PVT1 and FOXM1 promotes GC growth and invasion. **J** PVT1 can promote the expression of VEGFA and Slug by binding to STAT3 and ultimately promote angiogenesis and VM. **K** PVT1 promotes the expression of HIF-1α by targeting miR-186. **L** PVT1 promotes the expression of FGF2 and CD151 by targeting miR-152. **M** PVT1 promotes the expression of Snail by targeting miR-30a. **N** PVT1 promotes GC multidrug resistance by upregulating the ATP-dependent efflux pump P-gp by promoting the expression of MDR1 and MRP1

Li et al. [9] verified that PVT1 downregulation inhibits glucose transporter protein type 1 through miR-150-5p, regulates glucose metabolism in tumor cells, promotes invasion and migration of OSCC cells, and inhibits cell apoptosis (Fig. 2A). Wang et al. [74] found that PVT1 accelerated the development of EMT and led to tumor progression in patients with OSCC.

Cisplatin is a chemotherapeutic agent commonly used for the treatment of OSCC. Most patients with OSCC with poor prognosis have multiple drug resistance; however, the underlying mechanism remains unclear. Wang et al. [75] reported that PVT1 regulates cisplatin resistance in patients with OSCC through the miR-194-5p/HIF-1α signaling pathway (Fig. 2B).

These studies confirm that PVT1 is responsible for the occurrence, invasion, and drug resistance of OSCC cells.

#### PVT1 in esophageal cancer

EC causes 500,000 deaths annually, accounting for 5.5% of all cancer-related deaths worldwide. Esophageal squamous cell carcinoma (ESCC) is the most common type [76]. The latest data show that the incidence of EC is only 3.1% among all cancers, although its mortality rate is 5.5% [53]. ESCC is mainly associated with a poor prognosis because early detection is difficult. Most patients present with extensive metastases at the time of diagnosis [76]. Therefore, its pathogenesis should be further explored to improve the diagnosis and treatment of EC.

Yang et al. [10] reported that PVT1 might function as a ceRNA by targeting miR-186-5p to upregulate the oncogene PTTG1 (Fig. 2C). Zheng et al. [77] revealed that lncRNA PVT1 induces EMT by regulating the expression of EMT markers (E-cadherin, N-cadherin, and vimentin) (Fig. 2D). Li et al. [78] found that PVT1 upregulates LASP1 expression by targeting miR-203, thereby promoting ESCC progression (Fig. 2E). Shen et al. [79] demonstrated upregulated miR-145 and downregulated FSCN1 expression after PVT1 knockout, which inhibited ESCC cell invasion, migration, and survival (Fig. 2F). Xu et al. [80] discovered that silencing PVT1 might lead to decreased YAP1 expression through phosphorylated LATS1, thereby inhibiting tumor growth (Fig. 2G).

In summary, these studies indicate that PVT1 is involved in ESCC progression and acts as a diagnostic biomarker and therapeutic target.

#### PVT1 in gastric cancer

GC is the fourth most common cancer and the second most lethal malignant tumor worldwide. With the development of gastrointestinal endoscopy technology and GC drugs, GC death rates have decreased in recent years. However, advanced GC remains difficult to cure [1]. Identifying new biomarkers and therapeutic methods is an effective method for determining the pathogenesis of GC.

Recent studies have shown that PVT1 promotes GC migration, invasion, and lymph node metastasis (LNM) [81–83]. Kong et al. [8] reported that PVT1 negatively regulates P15/P16 through the epigenetic recruitment of EZH2, thereby promoting GC proliferation (Fig. 2H). Subsequently, Xu et al. [84] revealed that PVT1 promotes GC growth and invasion through a positive feedback loop with FOXM1 (Fig. 2I). In addition, Zhao et al. [43] found that PVT1 binds to STAT3, resulting in the recruitment of STAT3 to VEGFA and Slug promoters, whereas increased VEGFA led to angiogenesis and increased Slug-mediated GC vasculogenic mimicry network formation (Fig. 2J).

PVT1 can also regulate tumor progression through the lncRNA–miRNA–mRNA axis in patients with GC. Huang et al. [85] reported that PVT1 plays a key role in GC pathogenesis and may be a potential therapeutic target via miR-186/HIF-1α signaling (Fig. 2K). Similarly, Li et al. [86] indicated that PVT1 promotes GC growth and invasion by competitively binding to miR-152 to regulate CD151 and FGF2 (Fig. 2L). Furthermore, Wang et al. [87] demonstrated that PVT1 controls the EMT of GC via the miR-30a/Snail axis (Fig. 2M).

PVT1 is also involved in multidrug resistance in patients with GC. Zhang et al. [88] reported that PVT1 promotes the expression of adenosine triphosphate-dependent efflux pump P-glycoprotein protein by upregulating multidrug resistance 1 (MDR1) and multidrug resistance-related protein 1 (MRP1) expression, thereby leading to the occurrence of multidrug resistance (Fig. 2N).

Thus, PVT1 can be used as a biomarker for the diagnosis and prognosis of GC. High expression often represents deep infiltration; advanced tumor, node, metastasis (TNM) stages; and poor prognosis.

#### PVT1 in hepatocellular carcinoma

Although the incidence of HCC has gradually stabilized after rapid growth in recent decades, the HCC survival rate is only 20% [1]. Approximately one-quarter of patients with HCC have genetic mutations [89]. Therefore, the pathogenesis of HCC should be studied to identify novel targets and treatments.

Ding et al. [12] found that increased PVT1 often indicated poor prognosis and a high recurrence rate, which was closely related to alpha-fetoprotein levels. Yu et al. [90, 91] suggested that PVT1 can be combined with several other lncRNAs as a complementary diagnostic tool for HCC. Wang et al. [92] revealed that hepatitis B virus (HBV) infection promotes proliferation, development, and stem cell characteristics in HCC cells by enhancing the transforming growth factor beta (TGF-β)1/lncRNA-hPVT1/NOP2 signaling pathway, thereby accelerating the cell cycle. Similarly, Yan et al. [93] found that PVT1 upregulation was more common in early-onset than late-onset HCC after HBV infection. Subsequently, Jiang et al. [94] confirmed that PVT1 expression in HCC cells caused by HBV infection was significantly higher than that in HCC cells without HBV infection. Gou et al. [44] found that PVT1 interacts with EZH2 to downregulate miR-214 and promote the proliferation and invasion of HCC cells. Another research group revealed that PVT1 increases the stability of the MDM2 protein by enhancing EZH2 and inhibiting P53 protein expression to promote HCC cell proliferation [95]. Furthermore, Zhang et al. [96] speculated that PVT1 may play an important role in the occurrence and development of HCC by affecting the DLC1 and Hippo signaling pathways.

**Fig. 3:**
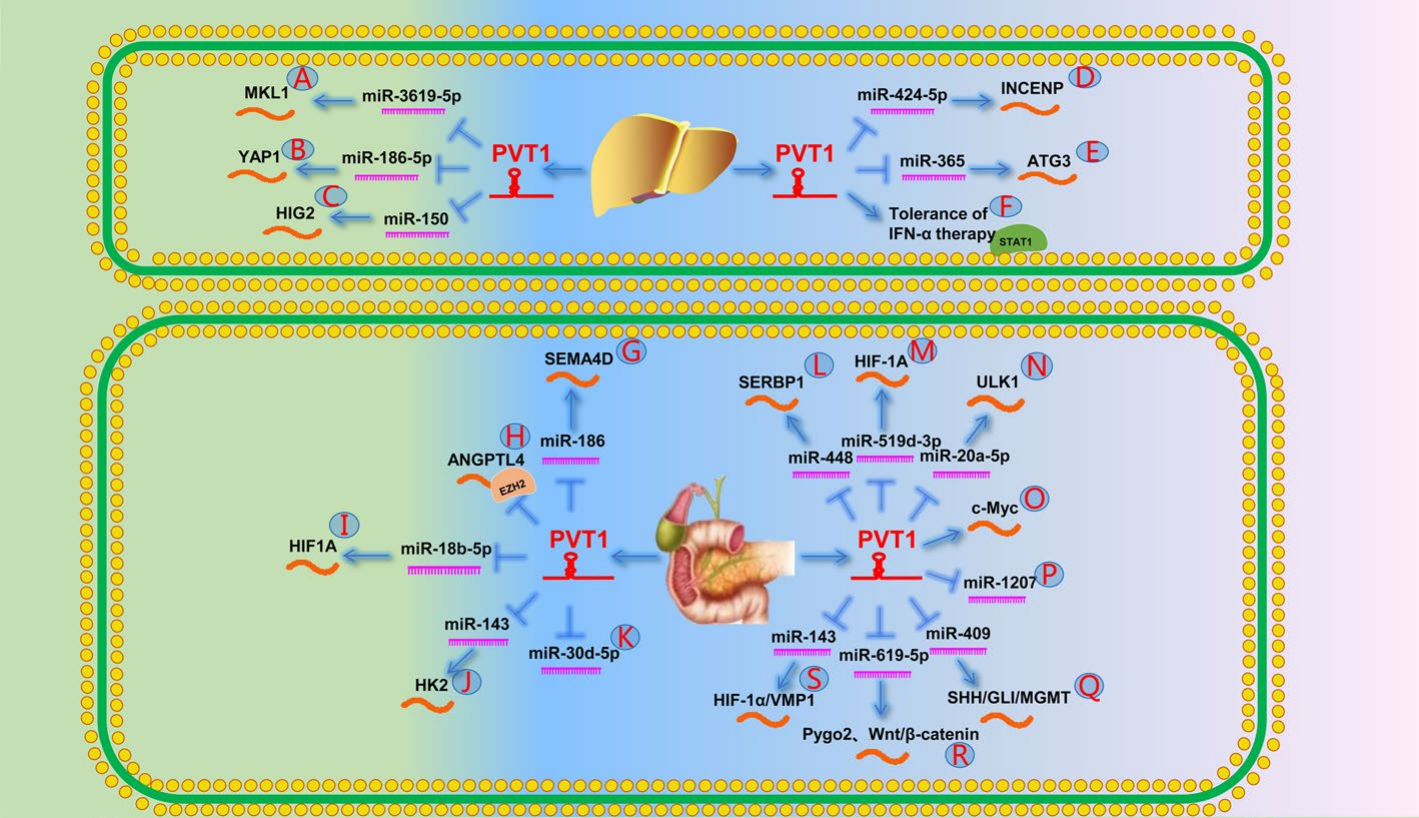
The role of PVT1 in the digestive system [2]. **A** PVT1 promotes the expression of MKL1 by targeting miR-3619-5p. **B** PVT1 promotes the expression of YAP1 by targeting miR-186-5p. **C** PVT1 promotes the expression of HIG2 by targeting miR-150. **D** PVT1 promotes the expression of INCENP by targeting miR-424-5p. **E** PVT1 promotes the expression of ATG3 by targeting miR-365. **F** PVT1 can decrease the phosphorylation of STAT1 and finally leads to the tolerance of IFN-α therapy. **G** PVT1 promotes the expression of SEMA4D by targeting miR-186. **H** PVT1 downregulates its expression by recruiting EZH2 to the ANGPTL4 promoter region. **I** PVT1 downregulates miR-18b-5p expression by binding to EZH2 and promotes HIF1A expression. **J** PVT1 promotes the expression of HK2 by targeting miR-143. **K** PVT1 directly binds to miR-30d-5p. (**L**) PVT1 promotes the expression of SERBP1 by targeting miR-448. **M** PVT1 promotes the expression of HIF-1A by targeting miR-519d-3p. **N** PVT1 promotes the expression of ULK1 by targeting miR-20a-5p. **O** PVT1 enhances gemcitabine resistance of PDAC by increasing c-Myc expression. **P** PVT1 enhances gemcitabine resistance of PC by inhibiting miR-1207 expression. **Q** PVT1 promotes the SHH/GLI/MGMT signal pathway by targeting miR-409. **R** PVT1 upregulates Pygo2 by targeting miR-619-5p and ultimately activates Wnt/β-catenin signaling pathway, resulting in PC resistance to gemcitabine chemotherapy. **S** PVT1 promotes the HIF-1α/VMP1 signaling pathway by targeting miR-143, resulting in PC resistance to gemcitabine chemotherapy

Increasing evidence shows that PVT1 functions as a ceRNA through lncRNA–miRNA–mRNA signaling. A previous study demonstrated that PVT1 upregulates the MKL1 expression by binding to miR-3619-5p in a positive feedback pathway. MKL1 in turn promotes PVT1 expression by binding to the CArG box, which is located in the PVT1 promoter region (Fig. 3A) [97]. Subsequently, several studies have shown that PVT1 promotes the invasion, migration, and proliferation of HCC cells by upregulating YAP1, HIG2, INCENP, and ATG3 by modulating miR-186-5p, miR-150, miR-424-5p, and miR-365 (Fig. 3B−E**)** [98–101], respectively.

Interferon alpha (IFN-α) has a significant effect on prolonging the postoperative disease-free survival and prognosis of patients with HCC, although patients’ tolerance to IFN-α remains an unsolved problem. A recent study suggests that IFN-α decreases PVT1 methylation by regulating H3K4me3 in the PVT1 promoter region, thereby increasing PVT1 expression. PVT1 subsequently reduces STAT1 phosphorylation, thereby blocking the IFN-α signaling pathway (Fig. 3F) [102].

The above evidence suggests that PVT1 plays an important role in the invasion, migration, proliferation, and drug resistance of HCC.

#### PVT1 in gallbladder cancer and cholangiocarcinoma

GBC is a rare disease that is caused by long-term gallstones and cholecystitis. The prognosis of GBC is usually poor because of early metastasis [103]. CCA is mainly derived from bile duct epithelial cells and peribiliary glands or hepatocytes. CCA is a fatal epithelial malignancy, and most patients have no identifiable risk factors [104]. Therefore, early detection of the related biomarkers can facilitate the early diagnosis of GBC and CCA, which is crucial for improving patient prognosis.

Yu et al. [14] showed that SRY-box transcription factor 2 (SOX2) activates the PVT1/miR-186/SEMA4D axis by promoting PVT1 expression, thereby enhancing CCA cell viability (Fig. 3G). Another study showed that PVT1 inactivated the ANGPTL4 promoter region by binding to EZH2, thereby regulating CCA vascular endothelial cell apoptosis (Fig. 3H) [45]. Similarly, Jin et al. [13] demonstrated that PVT1 recruits DNA methyltransferase through EZH2 and inhibits miR-18b-5p, thereby upregulating HIF-1α and promoting GBC cell proliferation (Fig. 3I). Moreover, Chen et al. [105] found that PVT1 controls aerobic glucose metabolism in GBC cells through the PVT1/miR-143/HK2 axis, thereby promoting tumor proliferation and metastasis (Fig. 3J). Liu et al. [106] reported that PVT1 promotes GBC progression by acting as a ceRNA of miR-30d-5p (Fig. 3K).

In conclusion, PVT1 shows strong carcinogenicity and can be used as a potential biomarker and therapeutic target in CBC and CCA.

#### PVT1 in pancreatic cancer

PC is the leading cause of cancer-related deaths worldwide, and is one of the top five causes of cancer-related deaths in the USA. According to the latest cancer statistics, PC has a 5-year survival rate of only 11%. Therefore, further exploration of the molecular markers is crucial for the early diagnosis and treatment of PC [1, 107].

Xie et al. [15] found that PVT1 and HOTAIR may serve as biomarkers for predicting early-stage PC. Two years later, Zhang et al. [108] demonstrated that PVT1 regulates PC cell survival, metastasis, and EMT by affecting the TGF-β/Smad signaling pathway. Similarly, Wu et al. [109] reported that silencing PVT1 increased tumor suppressor P21 and downregulated the EMT transcription factors Snail and ZEB1, suggesting that PVT1 affects EMT through P21 in PC cells. Moreover, Zhao et al. [110] showed that PVT1 promotes PC cell proliferation and migration by functioning as a ceRNA to regulate SERBP1 by sequestering miR-448 (Fig. 3L). Another study reported that PVT1 acts as a sponge for miR-519d-3p to modulate HIF-1α, which in turn activates angiogenesis, glucose metabolism, cell proliferation, migration, and metastasis (Fig. 3M) [111]. Subsequently, Zhu et al. [112] reported a positive feedback regulatory loop between PVT1 and HIF-1α, indicating that HIF-1α promotes PVT1 expression in hypoxia, whereas PVT1 promotes HIF-1α expression in normoxic environments. In addition, Huang et al. [113] revealed that PVT1 promotes the development of PC by regulating miR-20a-5p/ULK1 to increase cellular protective autophagy (Fig. 3N).

Chemotherapeutic drugs, such as gemcitabine (GEM), are important in the treatment of patients with advanced PC. Therefore, predicting the efficacy of chemotherapy and exploring the causes of chemotherapy resistance are important for improving patient prognosis and making correct treatment decisions. Wang et al. [114] pointed out that the combination of PVT1, HOTTIP, and MALAT1 may be a new noninvasive biomarker for predicting GEM efficacy in patients with PC. Another study showed that PVT1 and EZH2 combine to form a complex that promotes c-Myc expression and enhances PC resistance to GEM (Fig. 3O) [40]. Another study by Sun et al. [115] showed that HAT1 improves the resistance of PC to chemotherapy by promoting PVT1 expression and inhibiting EZH2 degradation. Moreover, You et al. [116] confirmed that the chemotherapy resistance of PC can be inhibited by silencing PVT1 or overexpressing miR-1207 (Fig. 3P). Furthermore, Shi et al. [117] showed that PVT1 can directly target miR-409 to promote the SHH/GLI/MGMT signaling pathway and enhance chemoresistance to GEM (Fig. 3Q). Similarly, Zhou et al. [118] found that PVT1 activates the Wnt/β-catenin signaling pathway via the miR-619-5p/Pygo2 axis to influence the chemotherapeutic sensitivity of GEM (Fig. 3R). A year later, Liu et al. [119] revealed that PVT1 knockdown inhibited autophagy by regulating the miR-143/HIF-1α/VMP1 axis, thereby improving PC cell sensitivity to chemotherapy (Fig. 3S).

Overall, PVT1 is strongly correlated with drug resistance in patients with PC and can be used as a new biomarker for PC.

#### PVT1 in colorectal cancer

CRC is the third most common cancer worldwide, with 151,030 new cases estimated to occur by 2022 [1]. CRC occurs at a younger age and is expected to become one of the leading causes of cancer-related deaths among people aged 20–49 years by 2030 [120, 121]. Developing more sensitive screening methods, enriching treatment options, and further exploring the role of PVT1 in CRC is urgently needed.

**Fig. 4:**
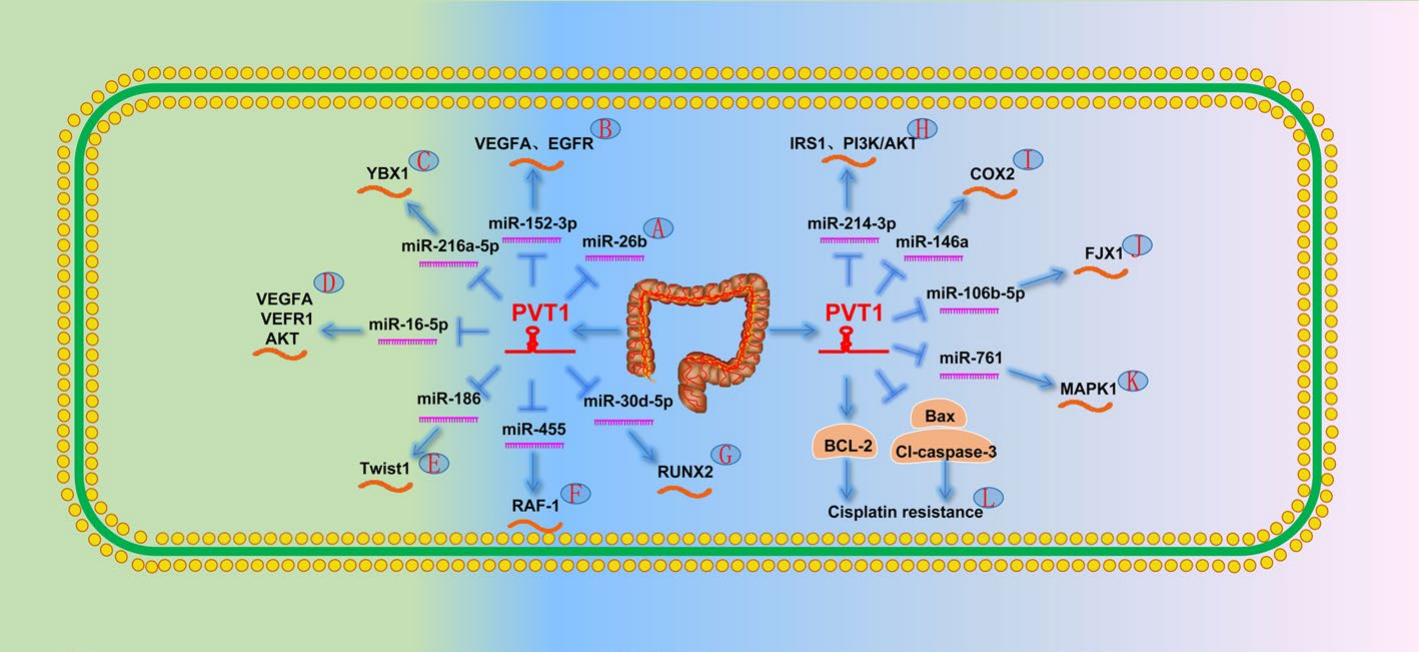
The role of PVT1 in the digestive system [3]. **A** PVT1 directly targets miR-26b. **B** PVT1 promotes the expression of VEGFA and EGFR by targeting miR-152-3p. **C** PVT1 promotes YBX1 expression by targeting miR-216a-5p and ultimately leads to EMT in CRC cells. **D** By targeting miR-16-5p, PVT1 leads to increased VEGFA, VEGFR1, and AKT expression and ultimately leads to EMT in CRC cells. **E** PVT1 ultimately promotes EMT in CRC cells by targeting miR-186 and leading to increased Twist1 expression. **F** PVT1 promotes RAF-1 by targeting miR-455. **G** PVT1 promotes the expression of RUNX2 by targeting miR-30d-5p. **H** PVT1 upregulates IRS1 by targeting miR-214-3p and ultimately activates PI3K/AKT signaling pathway. **I** PVT1 promotes the expression of COX2 by targeting miR-146a. **J** PVT1 promotes the expression of FJX1 by targeting miR-106b-5p. **K** PVT1 promotes the expression of MAPK1 by targeting miR-761. **L** PVT1 promotes the occurrence of EMT in CRC cells by downregulating the pro-apoptotic proteins Bax and Cl-caspase-3 and upregulating the expression of the anti-apoptotic protein BCL-2

PVT1 upregulation promotes the proliferation, invasion, and metastasis of CRC and suggests poor prognosis [48–51]. PVT1 is a highly effective biomarker for early CRC screening and has greater diagnostic and prognostic value than that of carcinoembryonic antigen [122, 123]. Zhang et al. [11] confirmed that PVT1 promotes CRC cell proliferation and metastasis by inhibiting miR-26b as an endogenous sponge (Fig. 4A). In addition, Guo et al. [124] reported that PVT1 in extracellular vesicles promotes CRC cell progression. Four years later, Lai et al. [125] demonstrated that PVT1 in exosomes upregulates VEGFA and EGFR by regulating miR-152-3p expression, thereby promoting the distant metastasis of CRC cells (Fig. 4B). In addition, Ansari and Shigeyasu et al. [37, 38] found that PVT1 extensively affects CRC signaling pathways, including the TGFβ/Smad and Wnt/β-catenin signaling pathways, and promotes CRC cell metastasis by regulating MYC, which may lead to a poor prognosis by promoting the ability of CRC stem cells.

Transformation of the cell phenotype from epithelial cells to mesenchymal cells is an important factor leading to tumor invasion and metastasis. YBX1 is a multifunctional tumor protein that promotes EMT by increasing the expression of angiogenesis factors. Zeng et al. [126] found that PVT1 promotes YBX1 expression by downregulating miR-216a-5p, thereby promoting EMT, invasion, and CRC cell metastasis (Fig. 4C). Recently, Wu et al. [127] found that PVT1 promotes angiogenesis and EMT through the miR-16-5p/VEGFA/VEGFR1/AKT signaling pathway (Fig. 4D). Radwan et al. [128] discovered a new signaling pathway that induces EMT. PVT1 increased the expression of the related transcription factor TWIST1 during EMT by downregulating miR-186 (Fig. 4E).

Chai et al. [129] reported that PVT1, as a key component of the RUNX2/PVT1/miR-455/RAF-1 axis, affects the growth and invasion of CRC (Fig. 4F). Subsequently, Yu et al. [130] confirmed that PVT1 promotes CRC cell proliferation through the miR-30d-5p/RUNX2 axis (Fig. 4G). Moreover, Shang et al. [131] reported that PVT1 upregulates IRS1 by targeting miR-214-3p and ultimately activates the PI3K/AKT signaling pathway to promote CRC proliferation and invasion (Fig. 4H). Several studies have reported on the lncRNA–miRNA–mRNA signaling in CRC and demonstrated that PVT1 promotes CRC cell proliferation, migration, and invasion by upregulating COX2, FJX1, and MAPK1 via the modulation of miR-146a, miR-106b-5p, and miR-761 (Fig. 4I−K) [132–134], respectively.

Chemotherapy is crucial in the treatment of CRC; however, the emergence of drug resistance often indicates a low survival rate and poor prognosis. Fan et al. [135] showed that PVT1 enhances cisplatin resistance in patients with CRC by inhibiting the endogenous apoptosis signaling pathway. Another research group discovered that PVT1 downregulated pro-apoptotic proteins [B-cell lymphoma 2 (Bcl-2) associated X protein and cleaved caspase-3] and upregulated the expression of anti-apoptotic protein BCL-2 (Fig. 4L) [136].

These findings indicate that PVT1 can be used as a new biomarker for the diagnosis and treatment of CRC, providing a novel target for future treatments.

### Role of PVT1 in the endocrine reproductive system

#### PVT1 in breast cancer

According to the 2022 Cancer Statistics, BC is the most common cancer among women, accounting for approximately 30% of all cancers. BC accounts for 15% of all cancer-related deaths and ranks second among the top ten causes of cancer-related deaths. Advances in BC diagnosis and treatment have greatly improved 5-year survival rates. However, recurrence, metastasis, and chemotherapy resistance remain urgent and unsolved problems [1]. Therefore, in-depth exploration of the molecular mechanisms can improve BC diagnosis in the future.

Guan et al. [16] showed that inhibition of PVT1 may inhibit BC proliferation. Zhang et al. [137] suggested that the GG genotype of the single nucleotide polymorphism rs13281615 may influence the development of BC through PVT1 and is associated with estrogen receptor positivity, higher tumor grades, and higher proliferative indices. Another study showed that activated PVT1-KLF5-CTNNB1 (β-catenin) promotes the proliferation of triple-negative BC [138]. RSPO1 is a key regulator of the β-catenin signaling pathway required for female development. A study showed that MYC-PVT1 overexpression causes RSPO1 upregulation, which ultimately leads to the development of cancer [139]. Another study showed that PVT1 promoted BC cell proliferation through the negative regulation of P21 [140]. A year later, Wang et al. [141] also demonstrated that PVT1 influenced EMT through P21, resulting in the proliferation and migration of triple-negative BC. The transcription factor SOX2 plays an important role in various cancers by controlling stem cell activity. Wang et al. [142] showed that PVT1 promotes EMT through SOX2 upregulation, thereby regulating BC invasion and growth. Zhu et al. [143] conducted a bioinformatics analysis and reported that a prediction model composed of four lncRNAs, PVT1, MAPT-AS1, LINC00667, and LINC00938, had good sensitivity and specificity for predicting BC prognosis. Subsequently, El-Fattah et al. [144] revealed the efficacy of PVT1, HOTAIR, NEAT1, PAI-1, and OPN as diagnostic biomarkers in BC. A year later, Levine et al. [145] suggested that PVT1 exon 9 may regulate BC cell migration by reducing claudin 4 expression.

**Fig. 5:**
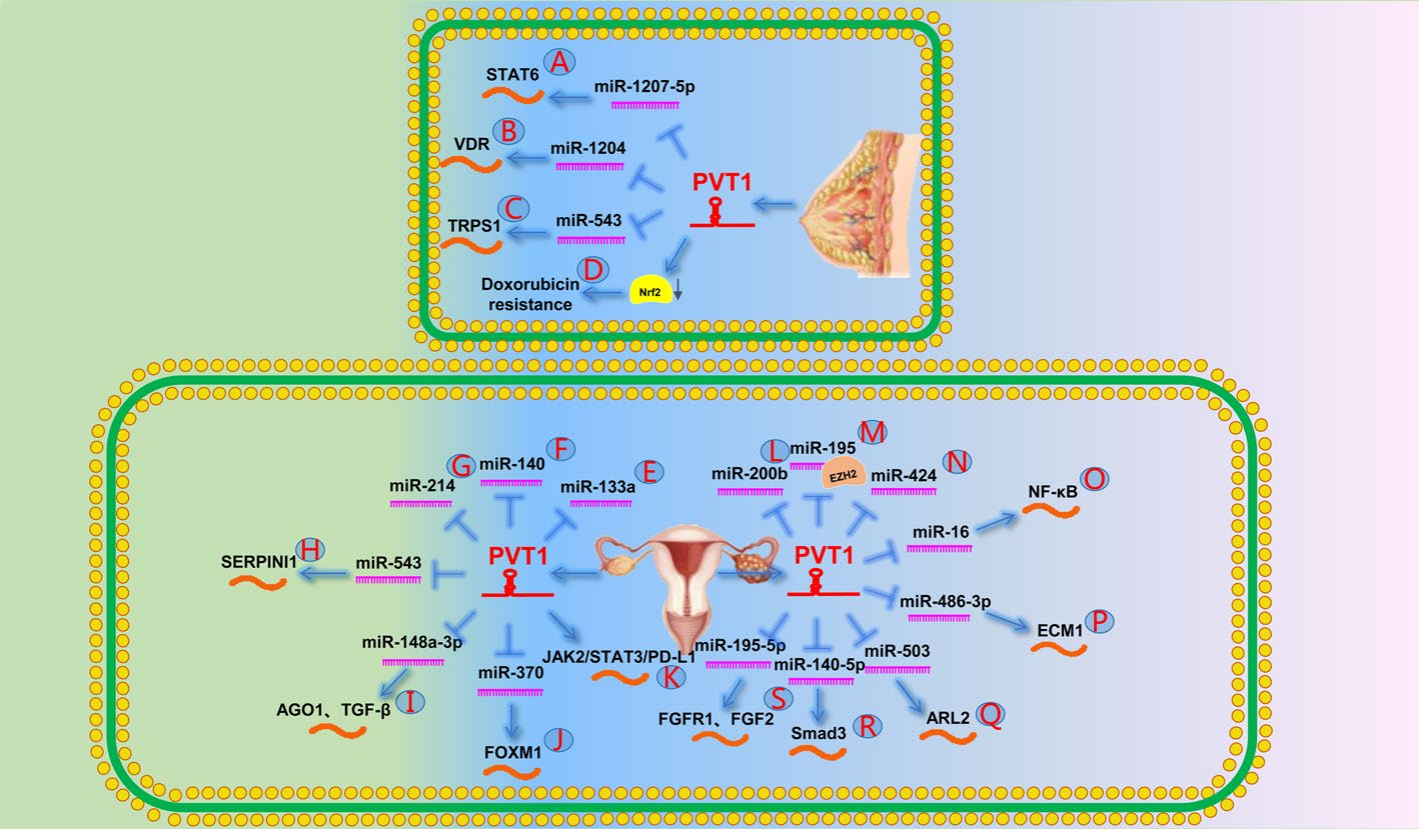
The role of PVT1 in the endocrine reproductive system. **A** PVT1 downregulates STAT6 by generating miR-1207-5p. **B** PVT1 targets VDR by generating miR-1204. **C** PVT1 promotes the expression of TRPS1 by targeting miR-543. **D** PVT1 caused Nrf2 degradation, eventually leading to doxorubicin resistance in TNBC. **E** PVT1 directly targets miR-133a. **F** PVT1 directly targets miR-140. **G** PVT1 directly targets miR-214. **H** PVT1 promotes SERPINI1 expression by targeting miR-543. **I** PVT1 upregulates AGO1 by targeting miR-148a-3p to promote the expression of TGF-β and ultimately leads to cisplatin resistance in OC. **J** PVT1 promotes FOXM1 expression by targeting miR-370. **K** PVT1 promotes OC cisplatin resistance by affecting the JAK2/STAT3/PD-L1 signaling pathway. **L** PVT1 directly targets miR-200b. **M** PVT1 directly targets miR-195. **N** PVT1 directly targets miR-424. **O** PVT1 promotes NF-κB signaling by targeting miR-16. **P** PVT1 promotes ECM1 expression by targeting miR-486-3p. **Q** PVT1 promotes ARL2 expression by targeting miR-503. **R** PVT1 promotes Smad3 expression by targeting miR-140-5p. **S** PVT1 promotes the expression of FGF2 and FGFR1 by targeting miR-195-5p

Moreover, Yan et al. [146] showed that PVT1 promotes BC cell proliferation and colony formation by downregulating STAT6 cell cycle regulators through miR-1207-5p (Fig. 5A). Similarly, Liu et al. [147] found that PVT1 promoted BC cell EMT and proliferation by increasing VDR by antagonizing miR-1204 (Fig. 5B). In addition, Wang et al. [148] reported that PVT1 affects cell proliferation by promoting TRPS1 expression by sponging miR-543 in BC cells (Fig. 5C).

In addition, PVT1 promoted chemotherapy resistance to adriamycin in triple-negative BC cells by competitively binding Keap1 to degrade the nuclear factor erythroid 2-related factor 2 protein (Fig. 5D) [149].

Taken together, these studies suggest that PVT1 plays an important role in the proliferation, invasion, and metastasis of BC, and is related to the drug resistance mechanism of triple-negative BC.

#### PVT1 in ovarian cancer

OC originates from the germinal epithelium of the ovary and can be divided into serous, endometrioid, clear cell, and mucous types. OC is the seventh most common cancer among women worldwide, with a survival rate of 46%. Therefore, the development of new detection methods and treatments is urgently needed [150].

Several studies have demonstrated that PVT1 regulates OC cell proliferation and invasion by acting as a ceRNA of miR-133a, miR-140, and miR-214 (Fig. 5E−G) [151–153]. Li et al. [154] demonstrated that PVT1 can inactivate EZH2 by recruiting it to the promoter region of tumor suppressor gene P57. Ketamine can reduce this recruitment, which in turn increases P57 expression. Another study showed that PVT1 knockdown may induce apoptosis through the miR-543/SERPINI1 axis in OC cells (Fig. 5H) [155].

PVT1 can regulate the resistance of OC to cisplatin by regulating the expression of apoptosis-related proteins such as TGF-β1, p-Smad4, and caspase-3 [19]. Wu et al. [156] demonstrated that PVT1 upregulates AGO1 by sponging miR-148a-3p, thereby downregulating TGF-β and promoting OC progression (Fig. 5I). Subsequent studies have also demonstrated that PVT1, TUG1, and MEG3 can lead to cisplatin resistance by inhibiting OC cell apoptosis [157]. Yi et al. [158] reported that PVT1 could directly bind to FOXM1 to stabilize its expression or promote FOXM1 expression by binding to miR-370 through a sponging effect, ultimately leading to OC progression and chemotherapy resistance (Fig. 5J). Another study reported that PVT1 silencing inhibits OC cell growth, metastasis, and cisplatin resistance by downregulating the JAK2/STAT3/programmed death-ligand (PD-L1) pathway. The authors suggested that combination therapy targeting PVT1 and the PD-L1 immune checkpoint blockade may have a synergistic effect on the clinical treatment of patients with OC (Fig. 5K) [159].

Martini et al. [160] analyzed the expression of multiple lncRNAs in 202 cases of early-onset OC and found that lncRNA PVT1 predicted a poor prognosis in patients with early-onset OC. Conversely, Yamamoto et al. [52] argued that high PVT1 and MYC expression predicted a good prognosis in patients with early-onset OC. High PVT1 and MYC expression may predict a good prognosis because of cell senescence induced by oncogenes and DNA damage caused by excessive replication stress in oncogenes. ATM-Chk1 and ATR-Chk2 pathway activation leads to p53 and retinoblastoma protein upregulation, which has a synergistic effect on cell growth arrest.

In conclusion, PVT1 may provide new insights for future treatment strategies and cisplatin resistance in OC. PVT1 may be a powerful biomarker for OC diagnosis and prognosis.

#### PVT1 in cervical cancer

CC is the second leading cause of cancer-related deaths among women aged 20–39 years [1]. Therefore, identifying the molecular markers and therapeutic targets is important.

Sun et al. [161, 162] showed that PVT1 could be used as a new non-invasive biomarker for the early diagnosis of CC. Iden et al. [17] showed that PVT1 may promote cell proliferation, migration, and invasion through interaction with nucleolar proteins, and is associated with poor prognosis of CC. Zhang et al. [46] revealed that PVT1 downregulates miR-200b by recruiting EZH2 to the miR-200b promoter region, thereby promoting CC cell cycle progression and migration (Fig. 5L). Subsequently, Shen et al. [163] confirmed that PVT1 attenuates miR-195 by binding EZH2 to increase the H3K27me3 level in the promoter region (Fig. 5M). They also found that PVT1 acts as a sponge to attract miR-195 and that PVT1 inhibition reduced paclitaxel-induced EMT and chemotherapy resistance. Moreover, Gao et al. [164] reported that PVT1 promotes the progression and invasion of CC by targeting miR-424 (Fig. 5N). Another study demonstrated that PVT1 promotes CC cell growth and invasion by downregulating TGF-β1 expression [165]. Further studies demonstrated that PVT1 regulated CC cell growth, invasion, and apoptosis by upregulating the nuclear factor-κB, ECM1, ARL2, and Smad3 pathways by modulating miR-16, miR-486-3p, miR-503, and miR-140-5p (Fig. 5O−R) [166–169], respectively.

These data demonstrate that PVT1 is an important molecular marker and significant therapeutic target for CC.

#### PVT1 in endometrial cancer

Endometrial cancer is the most common metastatic malignant tumor among women, and originates from endometrial epithelial cells [170]. Therefore, there is an urgent need to elucidate the underlying molecular mechanisms.

Kong et al. [18] demonstrated that the PVT1-miR-195-5p-FGFR1/FGF2 axis plays an important role in endometrial cancer (Fig. 5S). They found that cell proliferation, migration, and invasion were inhibited by PVT1 knockdown.

In conclusion, PVT1 plays a key role in endometrial cancer and is expected to become a new therapeutic target and be included in diagnostic screening methods in the future.

### Role of PVT1 in the urinary system

#### PVT1 in renal cell carcinoma

By 2022, 79,000 new cases of kidney and pelvic cancer will be diagnosed in the USA, and 13,920 people will die from the disease [1]. Approximately 85% of kidney tumors are RCC [171]. RCC is classified into three subtypes: clear cell RCC (ccRCC), papillary carcinoma, and chromophobe RCC. RCC is associated with late detection and poor prognosis; therefore, new and effective detection methods must be explored [172].

**Fig. 6:**
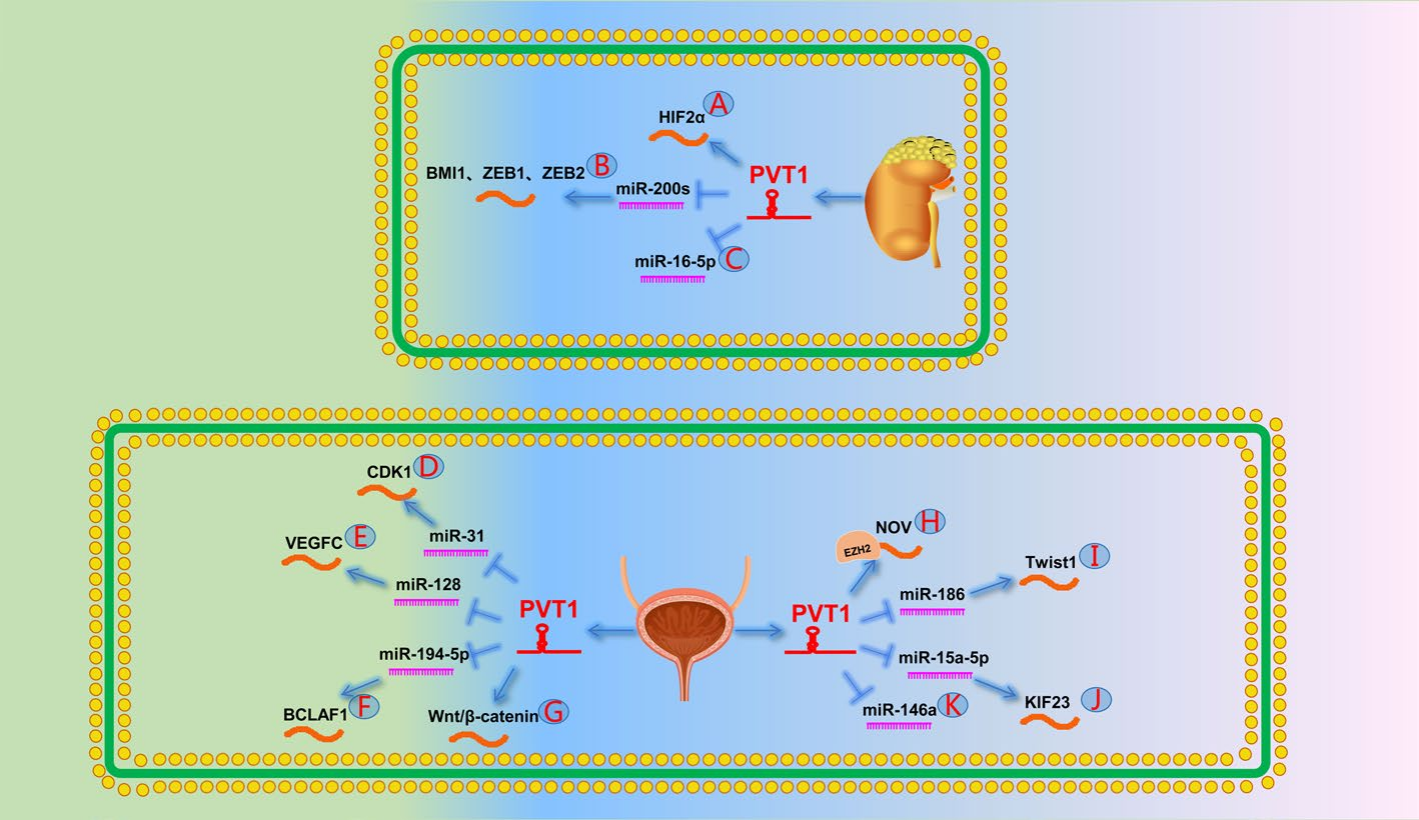
The role of PVT1 in the urinary system. **A** PVT1 forms a positive feedback loop with HIF2α protein. **B** PVT1 promotes BMI1, ZEB1, and ZEB2 by targeting miR-200s. **C** PVT1 can directly target miR-16-5p. **D** PVT1 promotes the expression of CDK1 by targeting miR-31. **E** PVT1 promotes the expression of VEGFC by targeting miR-128. **F** PVT1 promotes the expression of BCLAF1 by targeting miR-194-5p. **G** PVT1 leads to chemoresistance in BCa by activating the Wnt/β-catenin signaling pathway. **H** PVT1 represses NOV expression by recruiting EZH2 to the promoter region of NOV. **I** PVT1 promotes Twist1 expression by targeting miR-186 and ultimately leads to EMT. **J** PVT1 promotes the expression of KIF23 by targeting miR-15a-5p. **K** PVT1 can directly target miR-146a

One study pointed out that hypomethylation of the PVT1 promoter leads to its upregulation in patients with RCC. PVT1 upregulation stabilizes MYC expression, promotes tumor occurrence, and predicts a poor prognosis [20]. Subsequently, Li et al. [173] further demonstrated that PVT1 regulates the RCC cell cycle as well as apoptosis, and proliferation by upregulating EGFR expression and affecting its downstream proteins, AKT and MYC. Another study showed that PVT1 promotes RCC cell growth and inhibits RCC cell apoptosis by upregulating Mcl-1 [174]. Recently, Zhang et al. [175] found that HIF-2α promotes PVT1 expression by binding to the PVT1 enhancer; similarly, PVT1 can protect HIF-2α from ubiquitin-dependent degradation. This PVT1/HIF-2α loop promotes tumorigenesis and metastasis in RCC (Fig. 6A). Wang et al. [176] showed that PVT1 is significantly associated with a poor prognosis in patients with ccRCC. Furthermore, Wu et al. [177] found a 5-lncRNA signature, including lncRNA PANDAR, PVT1, LET, PTENP1, and linc00963, which was used to differentiate benign and malignant renal masses. Similarly, Liu et al. [178] proved that PVT1 combined with TCL6, mIR-155HG, and HAR1B could be used as biomarkers to evaluate the prognosis of ccRCC. Yang et al. [179] reported that PVT1 promotes the proliferation and metastasis of RCC by competitively binding miR-200s to upregulate BMI1, ZEB1, and ZEB2 (Fig. 6B). Another study showed that PVT1 acts as a sponge for miR-16-5p to regulate apoptosis and EMT in RCC cells (Fig. 6C) [180].

In summary, PVT1 was specifically upregulated in RCC, especially ccRCC. Many studies have shown that PVT1 combined with other lncRNAs is an efficient and accurate method for RCC diagnosis and prognosis prediction.

#### PVT1 in bladder cancer

Bladder cancer is the sixth most common cancer in men, with approximately 500,000 new cases and 200,000 deaths worldwide each year [181]. Current diagnostic markers, such as bladder tumor antigen and nuclear matrix protein, are not suitable for early bladder cancer owing to their lack of specificity and sensitivity [182]. Therefore, identifying new biomarkers is necessary for early diagnosis.

Zhuang et al. [21] reported that downregulation of PVT1 inhibits bladder cancer growth and induces apoptosis. Another study showed that elevated PVT1 was significantly associated with poor prognosis in muscle-invasive bladder cancer [183]. Tian et al. [184] demonstrated that the PVT1/miR-31/CDK1 pathway is responsible for the invasion, metastasis, and occurrence of bladder cancer (Fig. 6D). VEGFC is a lymphatic vasculature growth factor involved in lymphangiogenesis, angiogenesis, and regional lymphatic metastasis. Subsequently, Yu et al. [185] reported that PVT1 promotes the growth and metastasis of bladder cancer cells by upregulating VEGFC expression by acting as a ceRNA of miR-128 (Fig. 6E). Recently, Chen et al. [186] reported that PVT1 expression was significantly increased in bladder cancer. The PVT1/miR-194-5p/BCLAF1 axis was reportedly involved in the malignant progression and development of bladder cancer (Fig. 6F).

Another study indicated that PVT1 promotes the expression of MDR1 and MRP1 in bladder cancer by activating the Wnt/β-catenin pathway, ultimately reducing the response of bladder cancer cells to doxorubicin and cisplatin (Fig. 6G) [187].

In conclusion, PVT1 plays an important role in bladder cancer and is a new biomolecular marker that may be used as a new therapeutic target and diagnostic tool.

#### PVT1 in prostate cancer

According to the analysis of the 2022 Cancer Statistics, the incidence of PCa in American men ranked first among all cancers, accounting for 27% of all cases [1]. Although prostate-specific antigen can detect PCa early, recurrence in patients with advanced PCa is still an important factor that affects prognosis. Therefore, the underlying mechanisms of PCa need to be further explored.

A study suggested that exon 9 of PVT1 may be closely associated with invasive PCa in men of African descent [22]. Four years later, Pal et al. [188] demonstrated that exon 9 of PVT1 induced the proliferation and migration of prostate epithelial cells and was correlated with castration resistance after androgen deprivation therapy. A year later, Videira et al. [47] pointed out that PVT1 upregulation downregulated the expression of the androgen suppressor gene NOV through epigenetic modifications that recruited EZH2 to its promoter. This eventually led to the progression of androgen-independent castration-resistant PCa (Fig. 6H). Moreover, Yang et al. [189] reported that the knockdown of PVT1 significantly inhibited PCa cell growth, promoted apoptosis, and reduced c-Myc expression in cells. Subsequently, Chang et al. [190] found that PVT1 acts as a sponge of miR-186 to promote the expression of the EMT-related transcription factor TWIST1 (Fig. 6I). Wan et al. [191] showed that PVT1 downregulation inhibited PCa cell proliferation by reducing the phosphorylation of mitosis-related molecule P38. Another study discovered that PVT1 upregulated the expression of tumor metastasis-related protein NOP2 and promoted the invasion and metastasis of PCa [192]. Recently, Wu et al. [193] demonstrated that PVT1 promotes PCa progression by mediating the miR-15a-5p/KIF23 pathway (Fig. 6J). In addition, another study pointed out that PVT1 promotes tumor growth by regulating miR-146a methylation (Fig. 6K) [194].

In conclusion, high PVT1 expression plays an important role in promoting tumorigenesis in castrate-resistant prostate cancer and PCa.

### Role of PVT1 in the immune system

The immune system is important as it regulates the body’s immune responses and functions. It is composed of immune organs, cells, and molecules. Changes in the immune system may affect the occurrence and development of various diseases, including cancer. Therefore, studying the effect of PVT1 on the immune system is of considerable significance for the future treatment of tumors through immune regulation [195].

**Fig. 7:**
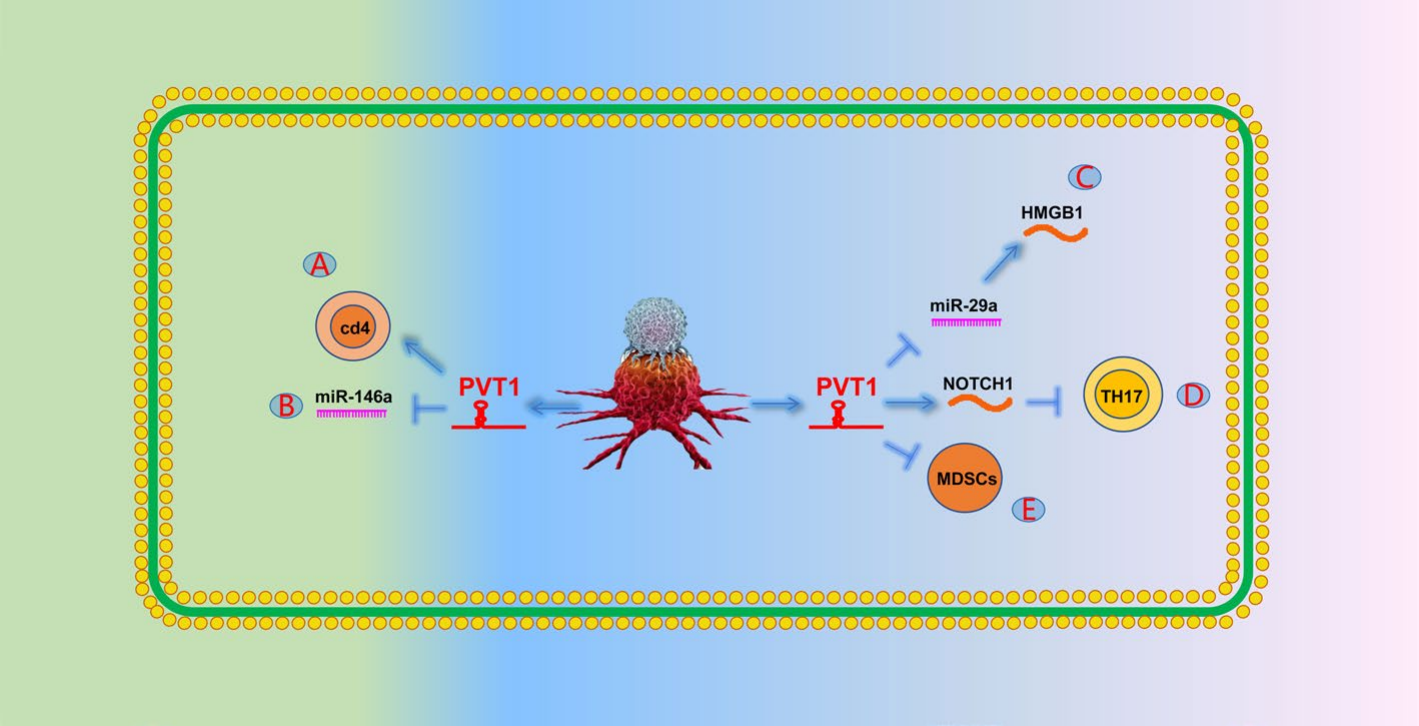
The role of PVT1 in the immune system. **A** PVT1 can activate CD4^+^ T cells. **B** PVT1 can promote autophagy of regulatory T cells by targeting miR-146a. **C** PVT1 can promote the expression of HMGB1 and promote the polarization of M1 macrophages by targeting miR-29a. **D** PVT1 can reduce TH17 differentiation by attenuating NOTCH1 expression. **E** PVT1 can promote the effect of MDSCs to achieve tumor immune suppression

Sjogren’s syndrome is a chronic autoimmune disease characterized by highly activated CD4^+^ T cells in the salivary glands. Fu et al. [196] found that PVT1 overexpression in patients with Sjogren’s syndrome promoted CD4^+^ T-cell activation (Fig. 7A). Lu et al. [197] showed that PVT1 promotes regulatory T-cell autophagy and ultimately inhibits allograft rejection by acting as a sponge for miR-146a (Fig. 7B). Luo et al. [198] reported that PVT1 promotes M1 macrophage polarization and aggravates myocardial injury through the miR-29a/HMGB1 axis in sepsis-induced myocardial injury. Immune thrombocytopenia (ITP) is an acquired autoimmune disease characterized by a significant increase in T helper 17 (Th17) cells in peripheral blood (Fig. 7C). Yu et al. [199] found that PVT1 overexpression reduced Th17 differentiation by downregulating NOTCH1 levels, thereby alleviating the occurrence of ITP. Myeloid-derived suppressor cells (MDSCs) can impair the antitumor response induced by T cells, thereby achieving tumor immunosuppression (Fig. 7D). Zheng et al. [200] found that the knockdown of PVT1 significantly inhibited the immunosuppressive ability of MDSCs (Fig. 7E).

### Role of PVT1 in tumors of other systems

#### PVT1 in glioma

Glioma is the most aggressive malignant tumor of the central nervous system and occurs mainly in the brain and glial tissues. The 5-year survival rate of glioma is only 0.05–4.7%. Therefore, exploring the pathogenesis of glioma is of great significance for improving its therapeutic effect in the future [201].

**Fig. 8:**
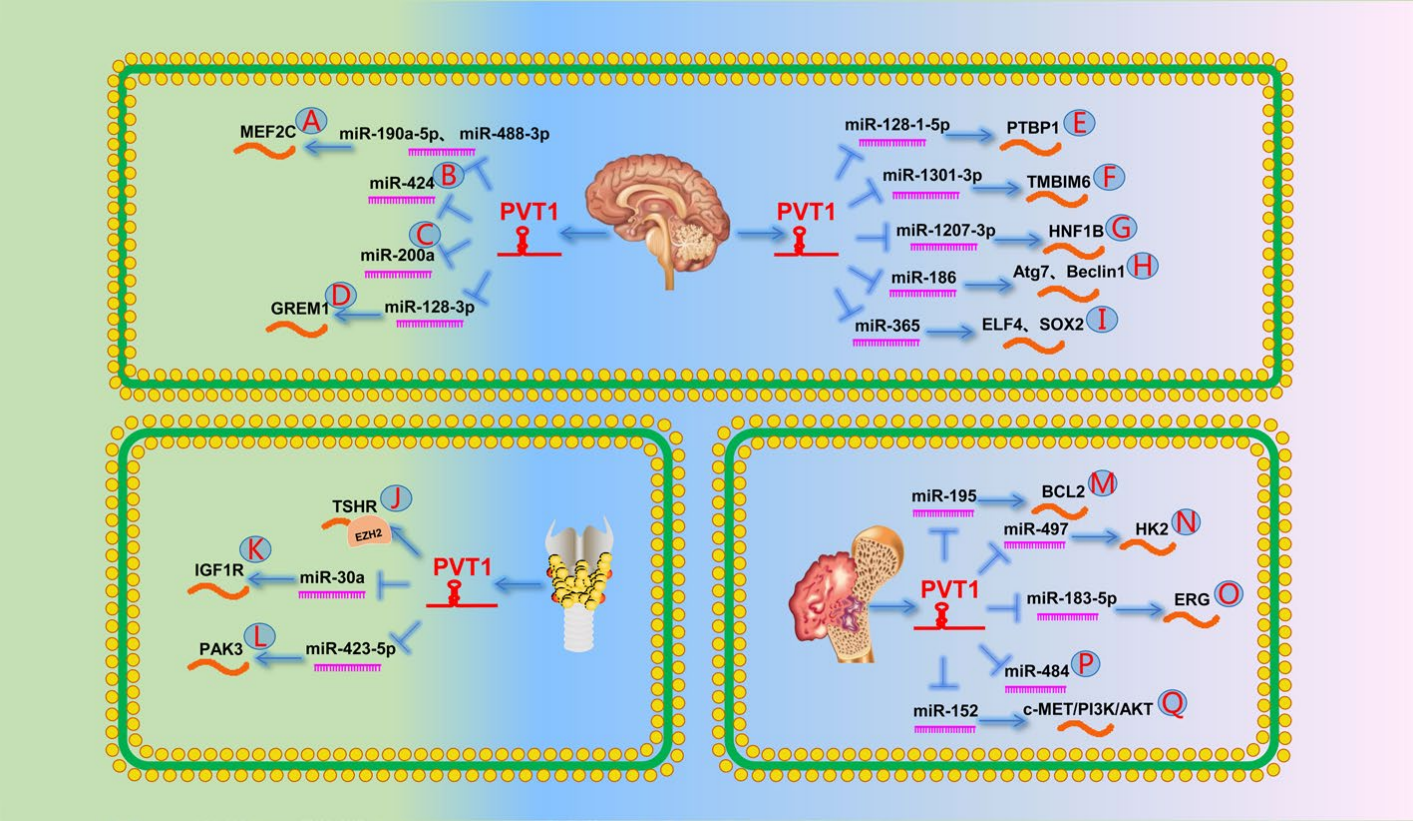
The role of PVT1 in the other systems. **A** PVT1 promotes the expression of MEF2C by targeting miR-190a-5p and miR-488-3p. **B** PVT1 can directly target miR-424. **C** PVT1 can directly target miR-200a. **D** PVT1 promotes the expression of GREM1 by targeting miR-128-3p. **E** PVT1 promotes PTBP1 by targeting miR-128-1-5p. **F** PVT1 promotes TMBIM6 by targeting miR-1301-3p. **G** PVT1 promotes HNF1B by targeting miR-1207-3p. **H** PVT1 promotes Atg7 and Beclin1 by targeting miR-186. **I** PVT1 promotes the expression of ELF4 by targeting miR-365 to regulate the expression of SOX2. **J** PVT1 represses its expression by recruiting EZH2 to the promoter region of TSHR. **K** PVT1 can directly target miR-30a. **L** PVT1 promotes the expression of PAK3 by targeting miR-423-5p. **M** PVT1 promotes the expression BCL2 by targeting miR-195. **N** PVT1 promotes the expression of HK2 by targeting miR-497. **O** PVT1 promotes the expression of ERG by targeting miR-183-5p. **P** PVT1 can directly target miR-484. **Q** PVT1 promotes the expression of the c-MET/PI3K/AKT signaling pathway by targeting miR-152, which leads to chemoresistance

One study showed that PVT1, as a ceRNA, binds to miR-190a-5p and miR-488-3p to upregulate myocyte enhancer factor 2C expression. It then promotes the expression of JAGGED1, which ultimately leads to increased malignant behavior in glioma cells (Fig. 8A) [202]. A year later, Han et al. [203] showed that PVT1 promotes glioma progression as a ceRNA of miR-424 (Fig. 8B). Another study came to a similar conclusion that PVT1 acted as a sponge of miR-200a and promoted glioma proliferation and invasion (Fig. 8C) [204]. A study by Lv et al. [205] indicated that PVT1 promotes glioma progression by downregulating UPF1 expression.

Many studies have revealed the important role of the lncRNA–miRNA–mRNA pathway in gliomas. Specifically, PVT1 reportedly promotes glioma cell proliferation, invasion, metastasis, and EMT by upregulating GREM1, PTBP1, TMBIM6, HNF1B, and Atg7/Beclin1 by modulating miR-128-3p, miR-128-1-5p, miR-1301-3p, miR-1207-3p, and miR-186, respectively (Fig. 8D–H) [23, 206–209].

PVT1 is also involved in chemotherapy resistance in glioma. Gong et al. [210] found that PVT1 upregulated ELF4 expression by acting as a ceRNA of miR-365, which then directly activated stem cell-related protein SOX2 expression, ultimately promoting stem cell characteristics and temozolomide resistance in gliomas (Fig. 8I).

Taken together, these studies demonstrate that PVT1 is an oncogene in glioma and may be a target for future therapy.

#### PVT1 in thyroid cancer

TC is the most common malignant tumor of the endocrine glands. According to the latest cancer statistics, an estimated 43,800 new cases were reported in 2022 [1]. Primary TCs include papillary TC (PTC), follicular TC, medullary TC, and anaplastic TC. PTC accounts for 85–90% of all TCs and has a good prognosis. Anaplastic TC is the most aggressive TC and has a poor prognosis and low survival rate [211]. Exploring the molecular mechanism of TC is of great significance for discovering new therapeutic targets in the future.

One study showed that PVT1 promotes TC cell proliferation by recruiting EZH2 and affecting TSHR expression (Fig. 8J) [26]. Another study showed that high PVT1 levels promoted TC cell growth and invasion by targeting the miR-30a/IGF1R axis and was associated with higher TNM and LNM stages (Fig. 8K) [212]. Two years later, Lin et al. [213] showed that PVT1 promoted malignant proliferation and migration of TC through the miR-423-5p/PAK3 pathway (Fig. 8L). In addition, Possieri et al. [214] showed that co-expression of MALAT1, PVT1, and HOTAIR could be used to distinguish benign and malignant thyroid nodules.

In conclusion, these results suggest that PVT1 plays a significant role in promoting TC, which is related to TNM and LNM stages, and TC tumor invasion, and can be used in the diagnosis of thyroid nodules.

#### PVT1 in osteosarcoma

OS is a rare tumor that often occurs in children and adolescents. Approximately 400 children and adolescents are diagnosed with OS in the USA every year, and as many as 25% of patients have metastasis by the time they are diagnosed. Therefore, exploring the mechanism of metastasis of OS is important for the development of future treatments [215, 216].

Zhou et al. [24] showed that PVT1 upregulates the expression of BCL2 by inhibiting miR-195, thereby inducing OS cell cycle arrest, inhibiting apoptosis, and promoting cell migration and invasion (Fig. 8M). Another study indicated that PVT1 upregulated HK2 by binding miR-497 and increasing glucose uptake and lactate production in OS cells, which ultimately accelerated cell proliferation and invasion (Fig. 8N) [217]. Similarly, Zhao et al. [218] showed that PVT1, as a ceRNA, inhibits its degradation and ubiquitination by binding to miR-183-5p, thereby upregulating ERG and ultimately promoting OS progression and metastasis (Fig. 8O). A year later, Yan et al. [219] found that PVT1 promoted metastasis in OS by targeting miR-484 (Fig. 8P). Xun et al. [220] demonstrated that PVT1 promotes EMT in OS cells and is highly correlated with TNM stage and distant metastases, which may be used as an independent prognostic risk marker of OS. In addition, Chen et al. [221] reported that the expression of PVT1 was upregulated in OS. Functionally, ALKBH5-mediated upregulation of PVT1 promotes OS cell growth in vitro and in vivo. Sun et al. [222] demonstrated that PVT1 enhanced the chemoresistance of OS by targeting miR-152 to activate the c-MET/PI3K/AKT signaling pathway (Fig. 8Q).

Taken together, these studies show that PVT1 is a significant factor in promoting OS progression and chemoresistance.

#### PVT1 in multiple myeloma

MM is a malignant tumor that originates from mutations in the bone marrow plasma cells and is characterized by abnormal proliferation and production of monoclonal immunoglobulins or light chains. Although current treatments can greatly improve remission rates, patients have a high risk of relapse or progression. Therefore, exploration of its pathogenesis is urgently needed to develop further treatments for this disease [223].

Yang et al. [25] demonstrated that PVT1 promotes MM cell proliferation by competitively binding to miR-203a. Handa et al. [224] showed that MYC and PVT1 synergistically promote MM progression.

Overall, these data suggest that PVT1 may serve as a future therapeutic target in patients with MM.

#### PVT1 in lymphoma

Lymphoma is a malignant tumor originating from lymphatic tissue and is mainly divided into Hodgkin’s and non-Hodgkin’s lymphoma (NHL). Hodgkin’s lymphoma is nearly twice as common as NHL, whereas the opposite is true for children with Hodgkin’s lymphoma [1]. Diffuse large B-cell lymphoma (DLBCL) is the most common and aggressive NHL subtype, accounting for 40% of NHL cases. Owing to the lack of effective treatments, survival rates remain very low [225].

Yang et al. [27] showed that overexpression of PVT1 promoted the proliferation of DLBCL cells and predicted poor prognosis.

This evidence suggests that PVT1 acts as an oncogene to promote lymphoma growth.

#### PVT1 in leukemia

Leukemia is a malignant tumor of the hematopoietic system. Leukemia can be classified as acute or chronic leukemia. Acute leukemia can be further classified as acute lymphoblastic leukemia (ALL) and acute myeloid leukemia (AML). Chronic leukemia can be classified as chronic myeloid leukemia and chronic lymphocyte leukemia (CLL). The 5-year survival rates are 27.4% and 84.2% for AML and CLL, respectively. Identifying new gene targets is important for improving leukemia cure rates [226].

Houshmand et al. [28] showed that PVT1 knockout in hematologic malignancies results in c-Myc degradation, decreased proliferation, increased apoptosis, and cell cycle arrest. PVT1 expression was higher in patients with AML than in healthy controls. In addition, PVT1 expression levels in the high-risk AML group were higher than in the medium- and low-risk AML groups. Similarly, Izadifard et al. [227] reported that PVT1 expression was higher in patients with AML than in healthy controls. PVT1 expression levels in the high-risk AML group were higher than in the medium- and low-risk AML groups. A subsequent study also confirmed that PVT1 and CCAT1 were highly expressed in patients with AML and were associated with poor prognosis [228]. Salehi et al. [229] showed that the inhibition of PVT1 significantly induced AML cell apoptosis and necrosis and reduced proliferation and C-MYC expression in AML cells. A year later, another study confirmed the relationship between PVT1 and c-Myc and suggested that the PVT1 blockade might be a potential treatment for AML [230]. In addition, Cheng et al. [231] found that PVT1 promotes the malignant progression of AML through the miR-29 family/WAVE1 axis, suggesting that PVT1 may be a potential therapeutic target for AML. Another study showed that PVT1 knockout significantly promoted apoptosis of ALL cells, blocked the cell cycle at G0/G1, reduced the proliferation rate, and downregulated the expression of oncogene c-Myc [232].

In conclusion, PVT1, as an oncogene, plays an important role in the development of leukemia and may be a therapeutic target in the future.

## Circular RNA PVT1 in various human tumors

The PVT1 gene located on chromosome 8 encodes lncRNA PVT1 as well as circular RNA PVT1 (circPVT1), obtained by circularization of its exon 2. circPVT1 is also a noncoding RNA that is widely present in the occurrence and development of various tumors [233].

In the respiratory system, circPVT1 has oncogenic effects in patients with NSCLC, lung adenocarcinoma, and lung squamous cell carcinoma (LSCC). Qin et al. [234] reported that circPVT1, as a ceRNA of miR-497, indirectly regulates Bcl-2 expression to promote the proliferation of NSCLC and inhibit cell apoptosis. Zheng et al. [235] demonstrated that circPVT1 regulates cisplatin and pemetrexed resistance in lung adenocarcinoma by acting as a sponge for miR-145-5p. Gao et al. [236] reported similar results, showing that circPVT1 enhances cisplatin resistance in lung adenocarcinoma cells by regulating miR-429/forkhead box K1 expression. Finally, Shi et al. [237] revealed that circPVT1 was significantly upregulated in the tissues, serum, and cell lines of patients with LSCC and promoted LSCC progression by increasing cyclin F expression, acting as a sponge for miR-30d and miR-30e.

In the digestive system, circPVT1 is closely related to the occurrence, development, and drug resistance of various tumors. He et al. [238] showed that circPVT1 regulates STAT3 levels by acting as a sponge for miR-125b and ultimately increases OSCC cell proliferation. Wang et al. [239] revealed that circPVT1 promotes OSCC progression by regulating the miR-143-3p/SLC7A11 axis and affecting the MAPK signaling pathway. Zhong et al. [240] found that circPVT1 regulated the progression of EC by influencing miR-4663 levels to modulate Paxs and peroxisome proliferator-activated receptor levels. Yao et al. [241] revealed that circPVT1 increased EC cell resistance to 5-fluorouracil by inhibiting ferroptosis through miR-30a-5p/Frizzled3. Chen et al. [242] found that circPVT1 was significantly upregulated in patients with GC and modulated the proliferation of GC cells by acting as a sponge for miR-125. These results suggested that circPVT1 expression is an independent prognostic marker. Liu et al. [243] showed that circPVT1 enhanced the resistance of GC cells to paclitaxel through the miR-124-3p/ZEB1 axis. Similarly, Wang et al. [244] showed that circPVT1 regulates the expression of HCC-derived growth factors by targeting miR-152-3p, leading to cisplatin resistance in GC cells. Zhu et al. [245] found that upregulation of circPVT1 regulates the proliferation and invasion ability of HCC cells by affecting the miR-203/homeobox D3 axis. Bu et al. [246] found that circPVT1 regulated HCC cell proliferation and glycolysis through sponging of miR-377. Wang et al. [247] found that circPVT1 regulated CRC cell metastasis by acting as a sponge for miR-145.

CircPVT1 also plays an important role in the endocrine and urinary systems [248]. Bian et al. [249] reported that circPVT1 regulated BC cell invasion and EMT by acting as a ceRNA of miR-204-5p. Wang et al. [250] revealed that circPVT1 promotes the ARG2/HIF-1α axis by regulating miR-29a-3p, thereby enhancing BC cell proliferation, invasion, and metastasis. In addition, Sun et al. [251] found that circPVT1 inhibited apoptosis and promoted OC cell proliferation by sponging miR-149. Zheng et al. [252] showed that circPVT1 affects ccRCC cell growth and metastasis by targeting the miR-145-5p/TX15 axis.

Leukemia is a disease that occurs mainly in the region of chromosome 8q24. Therefore, studying circPVT1, which is also located on chromosome 8, is of great significance for further exploration of the pathogenesis of leukemia [253]. Hu et al. [254] found that circPVT1 was significantly upregulated in patients with ALL and promoted cell proliferation by promoting the expression of c-Myc and Bcl-2. Gaffo et al. [255] showed that circPVT1 was significantly upregulated in children with B-precursor ALL. Moreover, a study by Chen et al. [256] revealed that circPVT1 was significantly upregulated in 68 patients with AML, and patients with high circPVT1 expression had significantly shortened overall and shortened event-free survival rates, suggesting that circPVT1 may be used as a biomarker to assess prognosis.

Osteosarcoma (OS) is a common primary bone malignancy. Noncoding RNA, as an oncogenic factor, also plays an important role in OS [257]. Wan et al. [258] demonstrated that circPVT1 inhibited miR-423-5p, thereby promoting the Wnt5a/Ror2 signaling pathway and affecting OS cell glycolysis, proliferation, migration, and metastasis. Huang et al. [259] found that circPVT1 inhibition significantly inhibited proliferation and induced apoptosis of OS cells through the miR-26b-5p/CCNB1 axis. Li et al. [260] validated that circPVT1 enhances the resistance of OS cells to doxorubicin by regulating the miR-137/TRIAP1 axis.

The abnormal expression of circPVT1 in patients with TC also warrants further investigation. Tao et al. [261] confirmed that circPVT1 inhibited PTC progression by sponging miR-126. Subsequently, Zeng et al. [262] showed that circPVT1 acted as a sponge for miR-195 to regulate VEGFA expression. circPVT1 promotes the Wnt/β-catenin signaling pathway and proliferation, migration, and invasion of PTC. In addition, Zheng et al. [261] reported that circPVT1 regulates EMT by targeting the miR-455-5p/CXCL12/CXCR4 signaling pathway and promotes thyroid medullary cancer cell proliferation, invasion, and migration.

## Conclusions

Cancer is one of the greatest risks to human health. By 2022, 1,918,030 new cases and 609,360 cancer-related deaths are estimated to occur in the USA [1]. Owing to delayed diagnosis, recurrence, metastasis, chemotherapy resistance, and other problems, patients tend to have poor prognosis and low survival rates. Therefore, the discovery of new biomarkers for early diagnosis and new targets in the treatment of cancer is vital for improving the survival rates of patients with cancer.

With the development of second-generation sequencing and bioinformatics, lncRNAs have gradually emerged in our field of vision, and have been found to play important roles in tumor progression. Previously, lncRNAs were reported to directly regulate chromatin topology or act as scaffolds for RNA and protein expression. Many studies have found that lncRNAs, including PVT1, are differentially expressed in tumor tissues compared with non-tumor tissues. However, the upstream and downstream regulatory mechanisms and how they affect the occurrence and development of tumors remain unclear. This study provides a new perspective for understanding the mechanisms underlying tumorigenesis and its development.

This review describes the mechanisms of action of PVT1 in various cancers. We found that PVT1 acts as a ceRNA to bind miRNAs and regulate downstream gene expression. PVT1 can also bind directly to the promoter region of the target gene, thereby promoting its methylation to regulate its expression. PVT1 also promotes the proliferation, invasion, and EMT of various tumor cells. For example, Wang et al. [87] pointed out that PVT1 promotes EMT in GC cells through the miR-30a/Snail axis. PVT1 is a biomarker that can be detected in serum. Wu et al. [177] constructed a model based on five lncRNAs (LET, PVT1, PANDAR, PTENP1, and linc00963), which are highly valuable in the diagnosis of ccRCC. In addition, PVT1 plays an important role in chemotherapy resistance. For example, tumor tissues with high PVT1 expression are resistant to GEM, cisplatin, adriamycin, and other chemotherapy drugs [40, 72, 149]. Some studies have also shown that PVT1 is a critical regulatory molecule in radiotherapy resistance. For instance, Wang et al. [70] showed that PVT1 significantly improved the radioresistance of NSCLC cells.

In summary, PVT1 plays an oncogenic role in many cancers. PVT1 is closely associated with the onset, development, invasion, migration, and treatment of cancer. PVT1 is an emerging biomarker for the diagnosis and treatment of cancer.

## Data Availability

Not applicable.

## Abbreviations

ALL: Acute lymphoblastic leukemia
AML: Acute myeloid leukemia
BC: Breast cancer
Bcl-2: B-cell lymphoma
CC: Cervical cancer
CCA: Cholangiocarcinoma
ccRCC: Clear cell renal cell carcinoma
ceRNA: Competitive endogenous RNA
circPVT1: Circular RNA PVT1
CLL: Chronic lymphocyte leukemia
CRC: Colorectal cancer
DLBCL: Diffuse large B-cell lymphoma
EC: Esophageal cancer
EMT: Epithelial–mesenchymal transition
ESCC: Esophageal squamous cell carcinoma
FGFR1: Fibroblast growth factor receptor 1
GBC: Gallbladder cancer
GC: Gastric cancer
GEM: Gemcitabine
HBV: Hepatitis B virus
HCC: Hepatocellular carcinoma
HIF: Hypoxia-inducible facto
IFN-α: Interferon alpha
ITP: Immune thrombocytopenia
lncRNAs: Long noncoding RNAs
LNM: Lymph node metastasis
LSCC: Lung squamous cell carcinoma
MDSCs: Myeloid-derived suppressor cells
miRNA: MicroRNA
MM: Multiple myeloma
MDR1: Multidrug resistance 1
MRP1: Multidrug resistance-related protein 1
mRNA: Messenger RNA
NHL: Non-Hodgkin’s lymphoma
NOTCH1: Neurogenic locus notch homolog protein 1
NPC: Nasopharyngeal carcinoma
NSCLC: Non-small cell lung cancer
OC: Ovarian cancer
OS: Osteosarcoma
OSCC: Oral squamous cell carcinoma
PI3K/AKT: Phosphatidylinositol 3-kinase/protein kinase B
PC: Pancreatic cancer
PCa: Prostate cancer
PTC: Papillary thyroid cancer
PVT1: Plasmacytoma variant translocation 1
RCC: Renal cell carcinoma
SOX2: SRY-box transcription factor 2
STAT: Signal transducer and activator of transcription
TC: Thyroid cancer
TGF-β: Transforming growth factor beta
Th17: T helper 17
TNM: Tumor, node, metastasis
VEGF: Vascular endothelial growth factor
